# Latest Update on lncRNA in Epithelial Ovarian Cancer—A Scoping Review

**DOI:** 10.3390/cells14070555

**Published:** 2025-04-07

**Authors:** Katarzyna Kwas, Maria Szubert, Jacek Radosław Wilczyński

**Affiliations:** Department of Surgical and Oncologic Gynaecology, 1st Department of Gynaecology and Obstetrics, Medical University of Lodz, 90-136 Łódź, Poland; maja.szubert@gmail.com (M.S.); jrwil@post.pl (J.R.W.)

**Keywords:** long noncoding RNA, epithelial ovarian cancer, molecular pathogenesis

## Abstract

Long noncoding RNAs (lncRNAs) are RNA molecules exceeding 200 nucleotides that do not encode proteins yet play critical roles in regulating gene expression at multiple levels, such as chromatin modification and transcription. These molecules are significantly engaged in cancer progression, development, metastasis, and chemoresistance. However, the function of lncRNAs in epithelial ovarian cancer (EOC) has not yet been thoroughly studied. EOC remains challenging due to its complex molecular pathogenesis, characterized by genetic and epigenetic alterations. Emerging evidence suggests that lncRNAs, such as XIST, H19, NEAT1, and MALAT1, are involved in EOC by modulating gene expression and signaling pathways, influencing processes like cell proliferation, invasion, migration, and chemoresistance. Despite extensive research, the precise mechanism of acting of lncRNAs in EOC pathogenesis and treatment resistance still needs to be fully understood, highlighting the need for further studies. This review aims to provide an updated overview of the current understanding of lncRNAs in EOC, emphasizing their potential as biomarkers and therapeutic targets. We point out the gaps in the knowledge regarding lncRNAs’ influence on epithelial ovarian cancer (EOC), deliberating on new possible research areas.

## 1. Introduction

### 1.1. Long Noncoding RNA (lncRNA)—Definition, Description

LncRNAs are a diverse class of RNA regulatory molecules that are longer than 200 nucleotides and do not encode proteins. Despite their lack of coding potential, lncRNAs are known to regulate gene expression at various levels, including chromatin modification, transcription, and post-transcriptional processing. These molecules can act as scaffolds, guiding chromatin-modifying complexes to specific genomic loci, or as decoys, sequestering transcription factors and other proteins. Additionally, lncRNAs form nuclear substructures and modulate mRNA stability and translation. Emerging research suggests that lncRNAs are pivotal in numerous biological processes and diseases and may serve as future biomarkers [1].

**Figure 1:**
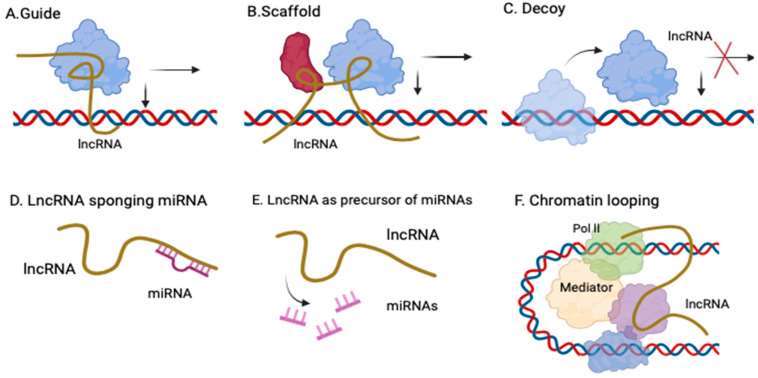
Regulatory mechanisms of lncRNA. (**A**). Guide lncRNA; (**B**)—scaffold lncRNA to promote RNP complexes; (**C**)—lncRNAs bind directly to protein molecules, impairing their functions; (**D**)—lncRNAs can act as ceRNAs (sponge), reducing effect upon the target mRNA, thereby regulating gene expression; (**E**)—lncRNA as miRNA precursor—some lncRNAs can act as precursors of miRNAs and modulate their activity; (**F**)—chromatin looping—lncRNAs serve as bridges to drive inter- or intra-chromosomal interactions.

Long noncoding RNAs (lncRNAs) can indeed act as competing endogenous RNAs (ceRNAs) by interacting with microRNAs (miRNAs) to regulate cell functions. This so-called sponging mechanism allows lncRNAs to sequester miRNAs, preventing them from binding to target mRNAs and thus modifying gene expression. MicroRNAs, small RNA molecules about 22 nucleotides long, typically bind to the 3′ untranslated regions (UTRs) of target mRNAs, either promoting mRNA degradation or inhibiting protein translation. Through this “sponging”, lncRNAs help control various cellular processes, including cell invasion, metastasis, and apoptosis. In addition to this sponge effect, lncRNAs participate in fundamental biological activities, such as genomic imprinting, X chromosome inactivation, chromatin modifications, and transcriptional regulation. Dysregulated lncRNA expression is thought to play a vital role in several diseases, including endometriosis. Studying expression patterns may uncover differentially expressed lncRNAs contributing to the disease’s pathogenesis, offering potential biomarkers or therapeutic targets. Identifying these lncRNAs provides valuable insight into the molecular mechanisms driving endometriosis and highlights their importance in disease progression and treatment development [1,2]. The main mechanisms of lncRNAs have been presented in Figure 1.

This review aims to present the latest update on lncRNA in EOC, describe the most studied lncRNA, and explain their possible place in the pathogenesis of EOC.

### 1.2. LncRNA—From the Discovery to the Role in Carcinogenesis

The advancement in sequencing technologies has revealed that over 80% of human DNA is transcribed into noncoding RNAs. In the early 1990s, the first lncRNAs were described, among them H19 and XIST. Since then, the amount of data on lncRNAs has been rising logarithmically. Firstly, their role was established as gene-specific regulatory molecules, and then our understanding expanded in the areas of embryogenesis and epigenetic regulation of allelic expression, such as the processes of dosage compensation and genomic imprinting. Lastly, they were proven to control pluripotency and influence lineage specification. Studies confirmed their impact on mammals’ neuronal development, various diseases, and carcinogenesis [3,4,5,6,7,8,9,10,11,12,13,14,15,16,17]. They have already been studied in many cancers, in in vitro studies on pathogenesis [9], but also on animal models, in which they affected tumor growth, immune modulation, survival, and chemoresistance, especially to platinum-based therapies [13,16,17]. There is also a lot of data on gynecological malignancies. In cervical cancer, MALAT1 has been engaged in cancer cell proliferation and migration by affecting epithelial–mesenchymal transition (EMT) and other signaling pathways [3]. MEG3 acted as tumor suppressors, inhibiting cancer cell growth and inducing apoptosis through their involvement in p53-mediated pathways [4,5].

In endometrial cancer, specific lncRNAs similarly act as oncogenes or tumor suppressors, contributing to cancer progression, invasion, and metastasis. LncRNA HOTAIR has been identified as an oncogenic factor, promoting tumor growth by altering gene expression and interacting with chromatin-modifying complexes [6]. GAS5 was found to be a tumor suppressor [7].

### 1.3. Epithelial Ovarian Cancer—Pathological and Molecular Background

Epithelial ovarian cancer is the third most common gynecologic neoplasm worldwide and the most prevalent form of ovarian cancer, accounting for approximately 90% of all ovarian tumors. [18,19]. The disease primarily affects postmenopausal women, with most diagnoses occurring in those aged 55 to 64. Risk factors include genetic mutations (notably *BRCA1* and *BRCA2*), family history of ovarian or breast cancer, age, nulliparity, early menarche, late menopause, and the use of hormone replacement therapy [20]. There have been various classifications of EOC according to histopathological subtypes, cancer aggressiveness, grading, and staging. For the actual review, we would like to familiarize readers with the WHO 2020 classification, which among serous tumors presents high-grade serous and low-grade serous cancers as separate entities: mucinous, endometrioid, clear cell, and undifferentiated carcinomas. These subtypes are differentiated based on their origin, microscopic appearance and biological behavior, prognosis, and the newest molecular findings, which, with high probability, will further influence this differentiation in the future [18,21,22].

Key genetic mutations implicated in ovarian carcinogenesis include *BRCA1* and *BRCA2* genes, which are involved in double-stranded break DNA repair mechanisms. Mutations in these genes disrupt homologous recombination, leading to genomic instability. Additionally, *TP53* mutations presented in over 96% of high-grade serous ovarian carcinomas contribute to defective cell cycle regulation and apoptosis. Oncogenic signaling pathways, such as PI3K/AKT/mTOR and RAS/RAF/MEK/ERK, are frequently dysregulated, promoting cellular proliferation, survival, and metastasis. Epigenetic modifications, including DNA methylation and histone acetylation, further influence gene expression profiles, often silencing tumor suppressor genes and activating oncogenes. The tumor microenvironment, comprising stromal cells, immune cells, and extracellular matrix components, also plays a critical role in ovarian cancer progression. For instance, interactions between cancer cells and the tumor microenvironment can facilitate angiogenesis, immune evasion, and chemoresistance [22,23,24]. Emerging evidence highlights the significance of noncoding RNAs, such as microRNAs and long noncoding RNAs (lncRNAs), in modulating gene expression and signaling pathways relevant to ovarian cancer. Understanding these molecular underpinnings is essential for developing targeted therapies and improving ovarian cancer diagnostic, prognostic, and therapeutic strategies. Current reports suggest that lncRNAs may be a potential target, as they influence the development and persistence of ovarian cancer through the modulation of inflammation, proliferation, angiogenesis, and tissue remodeling [24,25].

(a) *Endometriosis-associated ovarian cancer (EAOC)*

EAOC consists of ovarian endometrioid carcinoma (OEC) and ovarian clear cell carcinoma (OCCC) [25]. When examining tissue remodeling, one should remember that endometriosis is a benign condition that can become cancerous, with atypical endometriosis being an intermediate stage.
Despite numerous reports on EAOC, the precise mechanism of malignant transformation from endometriosis to ovarian cancer remains unclear. Kok et al. stated that endometriosis increased the risk of EOC fourfold [26]. A critical factor in the pathogenesis of EAOC is the *ARID1A* (AT-rich interactive domain 1A) suppressor gene, which encodes the BAF250a protein. The mutation rate of *ARID1A* in OCCC ranges from 40% to 95%, while in OEC, it is around 30%. It is confirmed as a first step in EAOC carcinogenesis [25]. Kristen rat sarcoma virus (*KRAS*) is another crucial gene involved in EAOC pathogenesis, with mutations identified in 29% of EAOC cases [27].

Additionally, oxidative stress and microRNAs are commonly implicated in the pathogenesis of EAOC. Recent reports suggest that lncRNAs may also be potential targets, as they influence the development and persistence of both ovarian cancer and endometriosis through the modulation of inflammation, proliferation, angiogenesis, and tissue remodeling. However, OCCC is a relatively rare cancer, and there is limited literature on the impact of lncRNAs on its pathogenesis. A similar situation exists for OEC; studies analyzing lncRNAs in their pathogenesis are scarce, with only two studies examining lncRNAs in OCCC [27,28,29]. Therefore, there is a considerable need for further studies that focus on the lncRNA role and expressions in OCCC and OEC.

(b) *High-grade serous ovarian cancer (HGSOC)*

HGSOC accounts for 70% of all ovarian cancers and is characterized by high-grade histology, rapid progression, poor prognosis, and high recurrence rate. Genetically, HGSOC is distinguished from low-grade EOC by frequent mutations in the TP53 gene, which are found in nearly all cases, along with other molecular alterations, such as BRCA1 and BRCA2 mutations. HGSOC is often diagnosed at an advanced stage due to its asymptomatic progression. Despite advances in surgery and chemotherapy, overall survival is still unsatisfactory. Platinum-based chemotherapy combined with paclitaxel remains the cornerstone of treatment but, unfortunately, is usually followed by chemo-resistant recurrence. However, recent advancements in targeted therapies, including PARP inhibitors and anti-angiogenic agents, like bevacizumab, have improved outcomes for some patients.

Nonetheless, the development of chemoresistance remains a significant challenge in managing HGSOC, necessitating ongoing research into novel therapeutic strategies and better biomarkers for early detection [30,31]. Analysis of lncRNA in HGSOC shows that dysregulated lncRNAs contribute to tumor development and chemoresistance. For instance, specific oncogenic lncRNAs, such as HOTAIR, have been shown to promote cancer cell invasion and metastasis by influencing epithelial–mesenchymal transition (EMT).

LncRNA MALAT1 has enhanced chemoresistance in HGSOC by interacting with these networks and promoting cell survival under stress. Moreover, some lncRNAs are associated with modulation of the tumor microenvironment, influencing immune evasion and angiogenesis, which are critical for tumor growth and dissemination. In contrast, tumor suppressor lncRNAs, like MEG3, may inhibit cell proliferation and induce apoptosis [32,33]. Due to their specificity and involvement in multiple cancer-related pathways, lncRNAs are emerging as potential biomarkers for diagnosis, prognosis, and therapeutic targets in HGSOC.

## 2. Material and Methods

**Figure 2:**
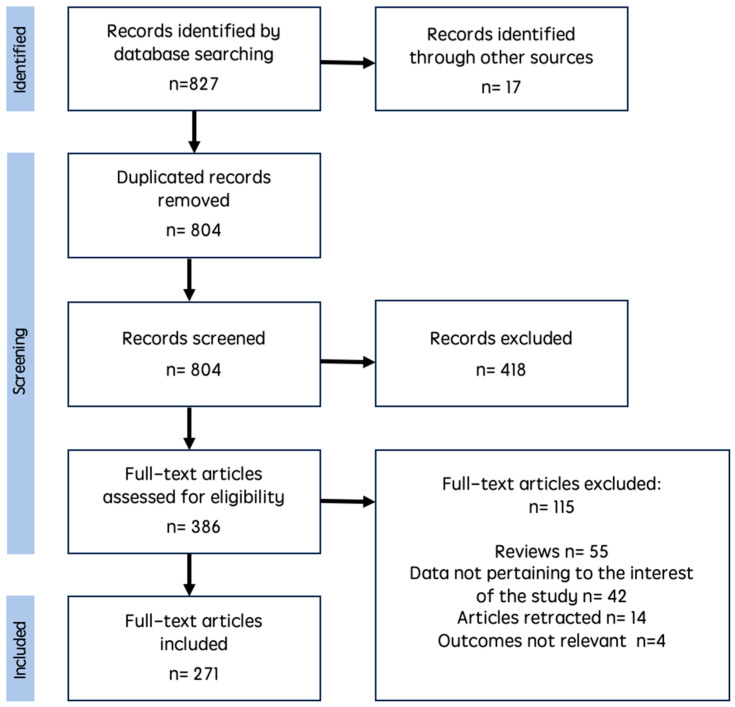
PRISMA flow diagram showing the research results from the study.

Since medical literature is rich in thousands of lncRNAs studies and gigabytes of data in many databases, we adopted PRISMA guidelines to search for scientific data regarding lncRNAs in the development of ovarian cancer [20]. We registered a search in the PROSPERO database (number: CRD42024611928) to systematize lncRNA data and its influence on survival in ovarian cancer patients. We followed typical steps for scoping and systematic review: identifying the research question, searching for relevant studies, study selection, charting the data, summarizing, and reporting results. We searched through Pubmed, Medline, Web of Science, SCOPUS, and ScienceDirect, using questions: “lncRNA in ovarian cancer”, “lncRNA AND ovarian carcinoma”, and “lncRNA AND epithelial ovarian cancer”. Two independent reviewers screened over 800 positions in databases. After exclusions (review and case report articles, full text unavailable, inappropriate methodology, data not possible to obtain, articles published before 1 January 2020), the last search was performed on 30 July 2024, and over 300 manuscripts were qualified for analysis. Figure 2 shows the results of the search according to the PRISMA 2020 guidelines [30]. According to the scoping nature of the review, no statistical tests were applied at this point of the analysis.

## 3. Results

We present a scoping review of the most important findings to give readers a broad overview of the available literature on a topic, identifying known evidence and gaps in knowledge. Summarizing the literature, Table 1 (downregulated lncRNAs) and Table 2 (upregulated lncRNAs) present the latest updates on lncRNAs and their functions in ovarian cancer. The lncRNAs are divided into two groups based on their expression levels in ovarian cancer, with a larger group of upregulated lncRNAs.

Presented lncRNAs are involved in many vital processes: cell cycle regulation, invasion, proliferation, migration, and apoptosis, and they promote epithelial-to-mesenchymal transition and resistance to chemotherapy. High expression levels of some lncRNAs are correlated with clinicopathological features such as tumor stage, FIGO classification, poor prognosis, and overall survival rate [79,89,119,147,152,240].

Among the upregulated lncRNAs, two major families are mostly presented: SNHG and LINC. The LINC family is the most prominent and widely studied, primarily involved in cancer cell migration, invasion, and proliferation, while inhibiting apoptosis through miRNA-mediated mechanisms, such as the miR-134-5p/TRIM44 and miRNA-4315p/SOX9 pathways [171,183]. Similarly, the SNHG family of lncRNAs (small nucleolar RNA host gene) regulates the cell cycle and targets various miRNAs like miR-139-5p and miR-98-5p, influencing oncoproteins. Notably, some SNHG members have specific roles in chemotherapeutic resistance, with SNHG7 regulating resistance to paclitaxel (PTX) and SNHG12 affecting sensitivity to carboplatin (CBDCA) [259,272,274].

The downregulated lncRNA group is smaller than the upregulated group. Decreased expression of these lncRNAs in ovarian cancer promotes cell cycle functions and, in some cases, contributes to chemotherapeutic resistance. They are also associated with the FIGO stage and histological type [35,36,58,77]. These downregulated lncRNAs target specific oncoproteins, such as PTEN and c-Myc, influencing cancer initiation and progression by regulating molecular pathways like PI3K/AKT/mTOR and Wnt/β-catenin.

## 4. Discussion

Analysis of particular groups of ovarian cancer showed scarcity in the literature regarding both HGSOC and EAOC cancers. Searching databases, we found not only well-conducted studies but also studies without a clear pathological description of ovarian cancer samples, which makes drawing conclusions about the appropriate type of ovarian cancer impossible. Nevertheless, some key findings regarding many lncRNAs are worth testing in in vivo studies or populations of different origins of ovarian cancer. Yuan et al. stated that upregulated NEAT1 influences cancer cell proliferation and angiogenesis by sponging miR-365 [215]. NEAT1’s role has also been confirmed by Liu et al. and proposed as a therapeutic target [213]. UCA1 has been determined to interact with the miR-654-5p/SIK2 axis and affect proliferation, migration, and invasion [293]; LINC01094 by sponging miR-577 has been stated to promote lymph node metastasis, cell proliferation, migration, invasion, and EMT [181]. Similar outcomes have been observed in the analysis of THOR, LINC01605, HCP5, and other lncRNAs presented in Table 1 and Table 2 [133,188,280]. Several lncRNAs have been suggested to impact the chemoresistance in HGSOC; upregulated HULC, RMRP, SNHG7, and UCA1 were shown to influence the PTX-resistance, SNHG12 was determined to affect the carboplatin (CBDCA) sensitivity, whereas CASC10, PLADE were described to promote cisplatin (CDDP) sensitivity [153,253,259,271,292].

Evaluation of lncRNAs in EAOC also showed a deficit in the literature since not many studies included data concerning these specific types of ovarian cancer. Upregulated DLX6-AS1 has been found to promote cancer cell proliferation, migration, and invasion by impairing apoptosis. Wu et al. suggested that PVT1 miR-148a/AGO1 interactions affected survival rate, metastasis, cell viability, and proliferation and correlated with the FIGO stage [110,240]. Similar results were obtained in the analysis of the NEAT1, XIST, GAS5, LINC01094, SNHG7, and many other lncRNAs, as presented in Table 1 and Table 2. Unfortunately, the influence of lncRNAs on chemoresistance in EAOC has not been yet studied.

Since we aimed to provide readers with the most up-to-date data, we discuss below the lncRNAs that have been studied most frequently and are the most promising factors for future analysis.

**The six most extensively studied lncRNAs in ovarian cancer.**

### 4.1. XIST

**Figure 3:**
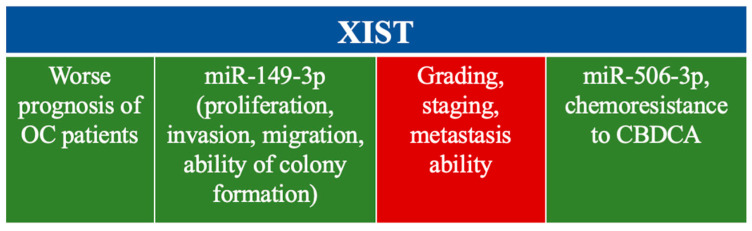
Summarizing of possible impact of XIST downregulated (red) and upregulated (green) in ovarian cancer in vitro studies.

The X inactivation-specific transcript (XIST) is the first long noncoding RNA (lncRNA) identified to play a role in the X chromosome inactivation. This lncRNA is dysregulated in various cancers, including liver, cervical, non-small cell lung, glioma, pancreatic, and breast cancers. The study by Jiang et al. demonstrated that lncRNA XIST is significantly upregulated in ovarian cancer (OC) tissues [301]. High expression of XIST was associated with poor prognosis of the patients. Moreover, Jiang et al. determined a negative relationship between XIST and miR-149-3p. Inhibition of XIST or promotion of miR-149-3p suppressed the proliferation, invasion, migration, and colony formation ability, thereby promoting apoptosis in vitro and limiting tumor growth in vivo [303]. Similar results were presented by Zuo et al., who stated that there was a significantly higher expression of XIST in EOC than in healthy ovarian epithelial tissue. Moreover, Zou et al. also studied the medical history of the patients. As stated, the expression of XIST was correlated with the tumor grade (*p* = 0.019), distant metastases (*p* = 0.021), and FIGO staging (*p* = 0.010). In further steps, the XIST expression was knocked down, and invasion and migration and the wound-healing ability of the EOC cells were determined to be reduced [304]. In the work of Xia et al., XIST expression and its association with miR-506-3p and FOXP1 in terms of (CBDCA) resistance in EOC were analyzed. XIST was upregulated in the EOC tissues, whereas miR-506-3p was downregulated. This overexpression of XIST was responsible for reduced apoptosis, and the result of XIST knockdown showed decreased cell autophagy, increased apoptosis, and resistance to CBDCA in EOC cells [298]. Finally, XIST was stated to positively regulate the FOXP1 expression sponging miR-506-3p, which showed the influence of this pathway on CBDCA resistance [305]. Interesting results were proposed by Wang et al., XIST was not only overexpressed in EOC, but its upregulation caused a significant increase in chemosensitivity. XIST was also associated with downregulated hsa-miR-214-3p. Moreover, the upregulation of hsa-miR-214-3p adversely regulated the anticancer effects of XIST overexpression in EOC [305]. According to Meng et al., XIST is involved in the regulation of tumor cell proliferation, invasion, and migration. Silencing XIST inhibits EOC cells’ proliferation, invasion, and migration by modulating the miR-335/BCL2L2 pathway [299]. Huang et al. identified XIST as a marker of chemotherapy response in ovarian cancer [300]. (Figure 3) Based on these data, a study on XIST expression as a blood marker in the population during chemotherapy could dispel doubts on its role as a prognostic factor for chemoresistance.

### 4.2. H19

**Figure 4:**
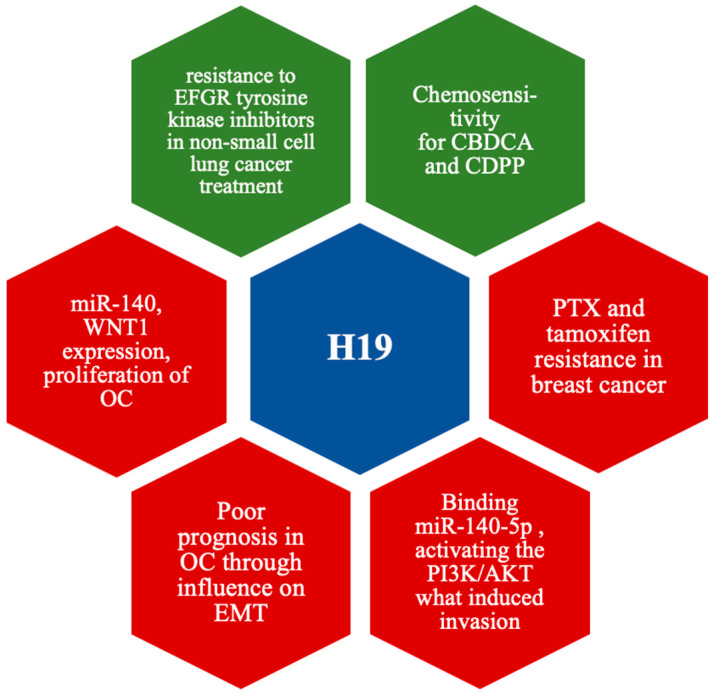
Possible mechanisms of action of upregulated H19 (red) and downregulated (green). Detailed description and abbreviations in text.

H19 expression was upregulated in most patients with breast cancer; what is more, H19 was described to be associated with paclitaxel (PTX) and tamoxifen resistance in breast cancer. On the other hand, the low expression of H19 was linked with resistance to EFGR tyrosine kinase inhibitors in non-small cell lung cancer treatment [305]. Xu et al. showed that H19 is responsible for multiple cellular processes in EOC. H19 was used to sponge the miR-140-5p, activating the PI3K/AKT signal pathway and inducing proliferation, invasion, migration, and epithelial–mesenchymal transition (EMT) in OC. However, the H19 was overexpressed, whereas miR-140-5p tended to have lower expression in OC cells. Furthermore, based on the ROC analysis, Xu et al. also suggested that H19 and miR-140-5p may potentially be effective markers for the diagnosis of OC; H19 diagnostic sensitivity was 72.73%, and specificity equaled 96.67% (*p* < 0.05). The results for miR-140-5p were 75.76 and 76.67%, respectively (*p* < 0.001) [128]. The study of Wang et al. presented comparable results, where H19 was highly expressed in ovarian cancer tissues; this overexpression of H19 was correlated with a poor prognosis of OC. Moreover, H19 acted as ceRNA to miR-140, upregulated the Wnt1 expression, and promoted the proliferation and migration of OC [127]. Li et al. stated the H19 role in EMT since, as described, H19 promotes the induction of TGF-B1 to EMT by acting as competing endogenous RNA for miR-370-3p in ovarian cancer cells [306]. Besides the H19 participation in many cellular processes, researchers also speculated on its role in CBDCA chemoresistance. The study of Tian et al. targeted assessing the chemoresistance to CBDCA in EOC. As indicated, H19 levels were significantly higher in EOC than in healthy ovarian tissue. Analysis of the chemoresistance determined that silencing CBDCA H19 in resistant EOC cells can impact carboplatin sensitivity. Therefore, H19 was stated to sponge the miR-29b-3p and target STAT3 (signal transducer and activator of transcription) [126]. Wu et al. made comparable conclusions, which indicated that the overexpression of H19 was strongly associated with resistance to cisplatin (CDDP) chemotherapy in OC. Similarly, the low expression of lncRNA H19 improved cisplatin sensitivity. Moreover, the overexpression of H19 was found to cause the increased migration and EMT activity of OC cells [307]. Unfortunately, despite the mentioned research, the literature is still insufficient to explain H19 in EOC; therefore, further studies are needed. The molecular mechanisms of H19 were presented in the Figure 4.

### 4.3. NEAT1

Another well-known lncRNA in the pathogenesis of ovarian cancer is nuclear paraspeckle assembly transcript 1 (NEAT1). Specifically in ovarian cancer, NEAT1 has been shown to interact with biological macromolecules, including chromatin, protein, and RNA, suggesting its potential involvement in regulating gene expression and cellular processes. The work of Xu et al. focused on the oncogenic role of the upregulated NEAT1 in ovarian cancer pathogenesis. Its knockdown significantly inhibited cell proliferation, invasion, and migration compared to the healthy ovarian tissue. Furthermore, a negative correlation was stated between the miR-4500 and NEAT1 expressions; this relationship influenced multiple cell functions, including cell proliferation, apoptosis, formation, glycolysis, etc. This study also described the BZW1 (basic leucine zipper and W2 domain-containing protein 1) as directly targeted by miR-4500 [216].

**Figure 5:**
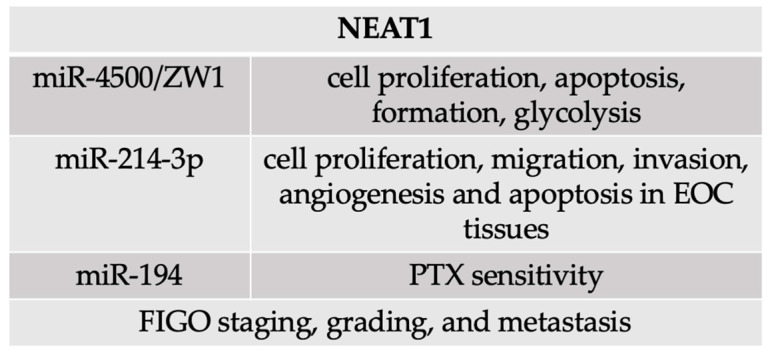
Summary of NEAT1 molecular mechanisms and features.

The regulatory effects of NEAT1 in human epithelial ovarian tissue were modulated via the miR-4500/BZW1 axis [216]. Similar results were obtained by Liu et al., who confirmed the high expression of NEAT1 in EOC and its contribution to its pathogenesis. In this study, researchers determined the association and regulation of miR-214-3p expression, identifying it as a target for NEAT1 sponging. Moreover, NEAT1 expression was responsible for enhanced cell proliferation, migration, invasion, angiogenesis, and reduced apoptosis in EOC tissues [212]. Chen et al. described the NEAT1 in ovarian cancer prognosis prediction. The upregulated NEAT1 was correlated with the FIGO staging, grade, and metastasis (*p* = 0.000); however, no association between the NEAT1 expression and tumor size was stated. Patients with higher expressions of NEAT1 had a statistically shorter overall survival rate than those with lower expressions. Finally, the multivariate analysis also determined that NEAT1 is a risk factor for the overall survival rate in patients with ovarian cancer [308]. Interesting findings were published by An et al., who studied the NEAT1 in paclitaxel (PTX) resistance. The significant upregulation of NEAT1 and downregulation of miR-194 was stated in patients resistant to PTX therapy. Moreover, NEAT1 was found to regulate the miR-194 expression negatively; thereby, the knockdown of NEAT1 caused the improvement of PTX sensitivity in patients resistant to such therapy and in ovarian cancer in vivo [309]. Similar results were proposed by Lou et al. [218]. Here, it is essential to underline the importance of the study of Yin at al., who used ovarian cancer tissues and ovarian cancer cell lines of different origins [217]. The summary of NEAT1 molecular features was proposed in the Figure 5.

### 4.4. MALAT1

Metastasis-associated lung adenocarcinoma transcript 1(MALAT1) has been broadly studied in various types of cancer, including ovarian cancer. MALAT1 is crucial in regulating gene expression and cellular processes, such as cell proliferation, migration, and invasion. According to multiple authors, MALAT1 functions as a ceRNA to regulate the expression of target genes by sponging and sequestering miRNAs [310,311]. Its function in ovarian cancer was broadly described by researchers who confirmed that MALAT1 facilitates epithelial ovarian cancer progression by cell proliferation, invasiveness, or migration. The work of Pei et al. showed that upregulated MALAT1 promotes cell proliferation, migration, and invasion in epithelial ovarian cancer cells by acting as a sponge for downregulated miR-22. MALAT1 also increased the EMT of EOC acting through the miR-ss/c-myc axis. Finally, the inhibition of MALAT1 caused the decline in vivo of EOC growth and its metastasis [201].

**Figure 6:**
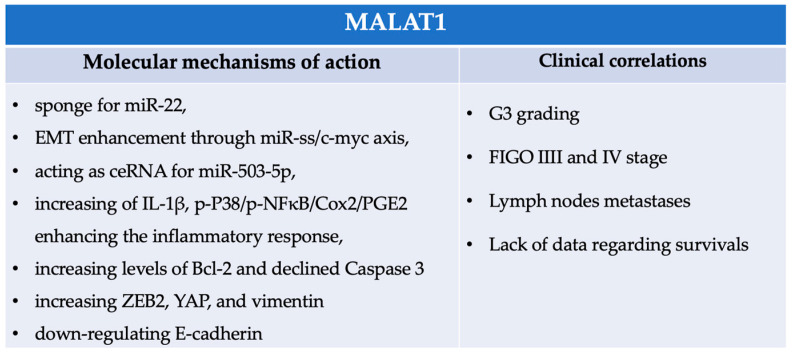
Summary of MALAT1 molecular mechanisms and clinical correlations.

Similar results were supported by Wu et al., who analyzed MALAT1 also in terms of clinicopathological characteristics, as it was reported that high MALAT1 expression was associated with advanced histological grade (G3, *p* < 0.001), higher FIGO stages (III-IV, *p* = 0.001), as well as the presence of lymph node metastases (*p* < 0.0001). Further analysis showed that the exogenous knockdown of MALAT1 inhibited the proliferation and migration of EOC, inducing apoptosis in EOC in vitro [310]. Likewise, Qiu et al. determined the high correlation between the serum exosomal, MALAT1 levels in EOC, and clinicopathological variables such as FIGO stage (*p* < 0.001) or lymph node metastases (*p* = 0.0020). Moreover, upregulated MALAT1 was determined to promote and regulate the angiogenesis in EOC [311]. Interesting results were published by Sun et al., who supported the idea that MALAT1 regulates EOC proliferation. In this study, upregulated MALAT1 correlated negatively, acting as ceRNA for miR-503-5p. Its knockdown resulted in an increased cell apoptosis inhibition as well as the inhibition of the JAK2/STAT3 signal pathway [59]. According to Mao et al., higher MALAT1 expression contributed to the resistance of PTX and CDDP in vitro. Overexpressed MALAT1 caused a significant increase in IL-1β, p-P38/p-NFκB/Cox2/PGE2 enhancing the inflammatory response. Increased levels of Bcl-2 and declined Caspase 3 levels were responsible for inhibiting cell apoptosis. In contrast, increased ZEB2, YAP, and vimentin with parallelly downregulated E-cadherin caused the amplification of EMT in EOC cells. This study also described that overexpression of MALAT1 in ovarian cancer-associated fibroblasts (CAFs) enhances the invasiveness of the cells [200]. In Figure 6, we summarized the MALAT1 molecular mechanisms. 

### 4.5. UCA1

The urothelial carcinoma associated 1 (UCA1) has been extensively studied in EOC, based on previous findings in various cancers, particularly urothelial carcinoma. Discovered as a tumor-associated RNA, UCA1 is overexpressed in several malignancies, contributing to tumor growth, metastasis, and chemoresistance. According to Qiu et al., the UCA1 expression was significantly higher in patients with EOC than in healthy ones. The upregulated UCA1 correlated with the FIGO stage (*p* = 0.005), histological grading (*p* = 0.000), peritoneal effusion (*p* = 0.00), as well as lymphatic metastases (*p* = 0.000), meaning that UCA1 may be involved in proliferation and invasion of ovarian cancer [312]. Furthermore, the expression of UCA1 was analyzed on epithelial ovarian cancer cell lines and epithelial cells from malignant ovarian cancer ascites; the results were comparable. Numerous studies investigated the UCA1 role in chemoresistance and chemotherapy in EOC cells. Li et al. described the upregulation of UCA1 in ovarian cancer cells and PTX resistance. Its knockdown caused the inhibition of PTX-resistant cell migration, invasion, and proliferation, suggesting the active role of UCA1 in regulating the PTX resistance in EOC cells. Furthermore, UCA1 expression was negatively correlated with the miR-654-5p in OC and PTX-resistant OC cells, indicating that UCA1 acts as a ceRNA for miR-654-5p. The authors concluded that UCA1 sponging the miR-654-5p also influenced the SIK2 protein expression, finally determining UCA1 in PTX resistance in EOC cells [293]. On the other hand, Wambecke et al.’s research determined that downregulation of the short isoform of UCA1 sensitized EOC cells to cisplatin by sponging miR-27a-5p, downregulating UBE2N, and regulating BIM protein expression, thereby increasing the sensitivity of cancer cells [292].

**Figure 7:**
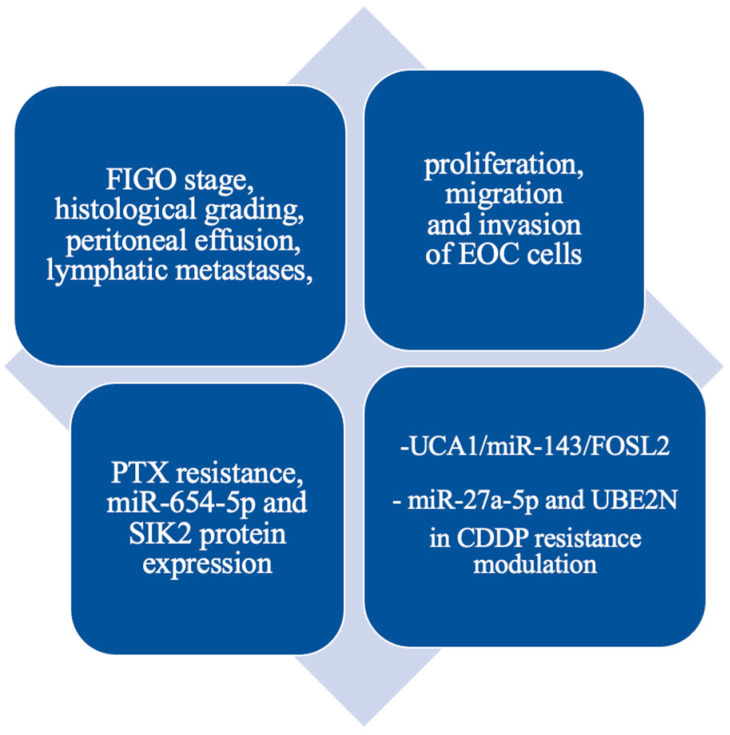
Summary of UCA1 molecular mechanisms and clinical features.

Similar results were proposed by Wang et al., and upregulated UCA1 was described to improve cell migration, invasion, and CDDP resistance. Moreover, SRPK1 kinase involvement in the effect of UCA1 was assessed, showing that inhibition of that kinase partially decreased the expression of UCA1 effect on EOC cells [313]. Another study showing the UCA1 role in CDDP resistance was presented by Li et al. In this study, researchers determined the subsequent pathway of the UCA1 mechanism, confirming the preceding results proposed by others. The upregulated UCA1 in EOC patients acts as ceRNA for miR-143 and targets FOSL2. The authors suggested that the UCA1/miR-143/FOSL2 pathway modulates the CDDP resistance and may be a potential therapeutic target for CDDP resistance in EOC patients [314]. The molecular mechanisms of UCA1 were summarized in Figure 7.

### 4.6. HOTAIR

**Figure 8:**
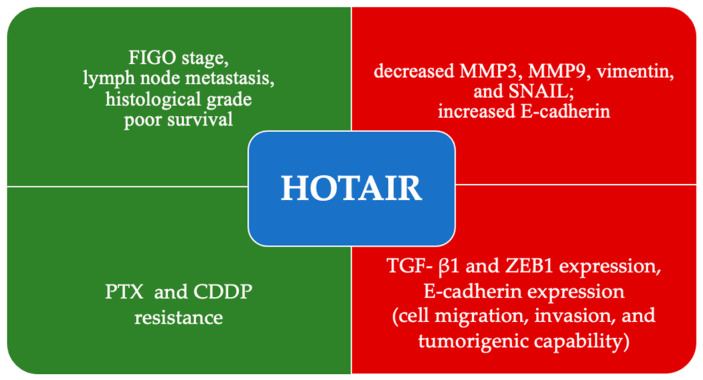
Mechanisms of action of downregulation of HOTAIR (red) and upregulation (green).

Another crucial lncRNA, broadly described in the literature, is the HOT antisense intergenic RNA (HOTAIR). This lncRNA was found to be highly upregulated in epithelial ovarian cancer tissues and cell lines, regulating various functions regarding tumor growth and chemoresistance, according to the work of Qiu et al. HOTAIR is significantly upregulated in EOC, and its expression is strongly associated with clinicopathological features, including histological grade (*p* = 0.009), FIGO stage (*p* < 0.001), and lymph node metastasis (*p* < 0.001). Furthermore, HOTAIR silencing inhibited migration, invasion in EOC in vitro, and metastasis in vivo. The knockdown decreased the expressions of MMP3, MMP9, vimentin, and SNAIL and increased E-cadherin, thereby suggesting the regulatory role of HOTAIR in EMT [315]. Zhou et al. also showed that HOTAIR was significantly expressed in EOC cells. On the other hand, downregulation of HOTAIR inhibited the TGF- β1 and ZEB1 expression, increasing the E-cadherin expression, thereby declining the EOC cells’ cell migration, invasion, and tumorigenic capability [138]. Significant results were presented by Jiang et al., who focused on PTX resistance in EOC; highly expressed HOTAIR was strongly associated with poor survival rate (HR 1.28 [1–1.65], *p* = 0.054) and significantly associated with PTX resistance. Knockdown of HOTAIR caused cell cycle arrest in phase G2/M, restoration of PTX sensitivity, and inhibition of ovarian cancer cell proliferation. Finally, HOTAIR was stated to regulate the checkpoint kinase (CHEK1), which could reinstate the PTX sensitivity [139]. A similar conclusion was made by Wang et al., who analyzed HOTAIR in terms of CDDP resistance. A significantly greater expression level in EOC cells resistant to CDDP than in nonresistant cells was stated. Moreover, the knockdown of HOTAIR inhibited cell migration and invasion and reestablished CDDP sensitivity in CDDP-resistant cells [316]. Other studies also found an association between HOTAIR expression and poor prognosis [316,317,318]. The molecular mechanism of HOTAIR was proposed in the Figure 8.

#### Major Concerns

Since lncRNAs do not encode proteins, their function and acting would be easy to establish. Also, their genomic context and attempts to classify them according to genetic location do not provide breaking information about their activity or evolutionary origin. Several of the above-mentioned lnRNAs have an increasing body of evidence from in vivo studies, but most lncRNAs await further verification. This verification from in vivo settings is of key importance if we consider implementing lncRNA into practice. First, Domcke et al. and then Croft et al. pointed out that a genomic comparison between cell lines and primary samples sometimes fails to recapitulate the primary disease subtype, especially when the microenvironment of the tumor, immune components, and microvessels are considered [319,320]. Among those provided in Table 2, 28 downregulated lncRNA have been found to influence chemoresistance to one or more chemotherapeutics. Six upregulated lncRNA have been described to either sensitize or cause resistance in in vitro studies. The following conclusion can be drawn: besides the appropriate in vitro cancerous models mirroring each type of ovarian cancer, the lncRNAs should be studied in groups potentially targeting one clinical question. These groups can be established with the help of AI tools, advanced screening of genomic databases, and different programs facilitating lncRNAs’ connection tracing.

## 5. Future Perspectives and Clinical Utility

LncRNAs are becoming promising therapeutic targets due to their high specificity in different cancer types and ability to regulate oncogenes and tumor suppressors. Advances in lncRNA-based therapeutics include the development of antisense oligonucleotides (ASOs), small interfering RNAs (siRNAs), and CRISPR-based systems aimed at silencing oncogenic lncRNAs or restoring the function of tumor-suppressive lncRNAs. Additionally, lncRNAs are being explored as biomarkers for early cancer detection and prognosis, offering a more precise approach to personalizing treatment.

In prostate cancer, lncRNAs such as SChLAP1, lncRNA-p21, and PCA3 have demonstrated clinical utility. PCA3 has been linked to the survival of prostate tumor cells by influencing the androgen receptor signaling pathway. It also plays a role in regulating the EMT by modulation of key targets like E-cadherin and TWIST. Additionally, PCA3 is incorporated into the PROGENSA gene signature, which helps to identify patients who, despite a previous negative biopsy, may require a second one. PCA3 has been FDA-approved for reducing unnecessary biopsies. Moreover, the integration of lncRNAs with other molecular markers enhances diagnostic accuracy, as seen with the Mi Prostate Score test, which combines PCA3, PSA, and TMPRSS2-ERG detection, achieving high sensitivity [321].

LncRNA-based biomarkers in breast cancer remain underexplored. Studies indicate their potential in patient stratification based on hormone receptor expression, with promising lncRNAs like NEAT1, DSCAM-AS1, and GATA3-AS1.

The stability of lncRNAs in biological fluids (e.g., blood, urine, saliva) positions them as valuable prospects for non-invasive cancer diagnostics. Several lncRNAs, including HOTAIR, H19, and MALAT1, have been successfully detected in liquid biopsies for breast cancer, supporting their potential as circulating biomarkers. Advanced molecular techniques, such as ISH-RNA and spatial transcriptomics, have further enhanced the sensitivity and specificity of lncRNA detection. Additionally, the integration of machine learning and molecular imaging methodologies may refine oncological diagnostics and personalized treatment strategies [321]. However, further research and clinical validation are required to establish lncRNAs as routine biomarkers for cancer management.

## 6. Conclusions

Long noncoding RNAs are emerging as crucial therapeutic targets in ovarian cancer due to their regulatory roles in tumor growth, metastasis, and treatment resistance. These RNA molecules influence key oncogenic pathways such as MAPK signaling, EMT, and vasculogenic mimicry, making them potential candidates for precision medicine.

As the molecular functions of lncRNAs become better understood, integrating them into existing therapies, such as combining lncRNA-targeting agents with immune checkpoint inhibitors or chemotherapy, could enhance treatment efficacy. Ongoing research into improving lncRNA therapeutics’ stability and targeting mechanisms could lead to groundbreaking treatments, transforming cancer therapy into a more targeted, less toxic approach shortly. Moreover, lncRNAs could serve as predictive biomarkers, helping tailor the chemotherapy regimens based on the lncRNA profiles of individual tumors and leading to more personalized treatment approaches. As research advances, combining lncRNA-targeting therapies with conventional chemotherapy or novel agents, like PARP inhibitors or immune checkpoint inhibitors, could offer synergistic effects, overcoming resistance and enhancing patient outcomes. Taking it all together, further research is needed to thoroughly analyze lncRNAs mechanisms in the context of potential markers and therapeutic targets in all types of ovarian cancer.

## Figures and Tables

**Table 1:** Downregulated lncRNAs in epithelial ovarian cancer. (+) increased, (−) decreased, ECM—extracellular matrix, EMT—epithelial–mesenchymal transition, PTX—paclitaxel, CDDP—cisplatin. All the studies included in the table were published after 2020.

Type of Ovarian Cancer	LncRNA	Target	Functions	Reference
EOC	AOC4P		FIGO stage, migration, invasion (+)	Lin et al. [33]
EOC	ASMTL-AS1	miR-1228-3p	FIGO stage, ascites cytology, lymph node (+) proliferation, migration, invasion (−)	Xu et al. [34]
EOC	CTBP1-AS2	miR-216a/PTEN	poor survival (+)	Cui et al. [35]
EOC	EPB41L4A-AS2	miR-103a	cell proliferation, migration, invasion (+)	Sun et al. [36]
EOC	FAM225B	DDX17/PDIA4	progression, migration, invasion, apoptosis (+)	Yao et al. [37]
EOC HGSOC	FGD5-AS1	miR-107/RBBP6	proliferation, angiogenesis (+)	Zhang et al. [38]
EOC OCCC	GAS5	miR-96-5p/PTEN	proliferation, invasion	Dong et al. [39]
		miR-23a/WT1	migration, proliferation, poor prognosis (+) apoptosis (−)	Zhou et al. [40]
		miR-31-5p/ARID1A	viability, invasion (+)	Zhang et al. [41]
		PI3K/AKT/mTOR pathway, hnRNPK	low survival, migration, invasion, proliferation (+) apoptosis (−)	
EOC	GAS8-AS1		viability, migration, invasion (−)	Fang et al. [42]
EOC, HGSOC	HAND2-AS1		viability, migration (−)	Gokulnath et al. [43]
		miR106a/PTEN	CDDP-resistance (−)	Li et al. [44]
EOC	HCG11	miR-1270/PTEN; AKT/mTOR pathway	proliferation, migration, EMT (−)	Chen et al. [45]
EOC	HOTAIRM1	miR-106a-5p, ARHGAP24	proliferation, invasion (+) apoptosis (−)	Chao et al. [46]
EOC	LEMD1-AS1		OS, FIGO stage (−)	Yang et al. [47]
		miR-183-5p/TP53	proliferation, migration, invasion (+)	Guo et al. [48]
EOC	LIFR-AS1		tumor size, grade, metastasis, poor prognosis (+)	Liu et al. [49]
EOC	LINC-PINT	miR-374a-5p	apoptosis (−) proliferation, migration, invasion, EMT (+)	Hao et al. [50]
EOC, HGSOC	LINC00629	LINC00629/c-Myc	proliferation, poor survival (+)	Liu et al. [51]
EOC	LINC00936	miR-221-3p	proliferation, migration, invasion, angiogenesis	Shu et al. [52]
EOC	LINC01296	miR-29c-3p	proliferation, invasion and migration (+)	Xu et al. [53]
EOC	LINC01508	Hippo/YAP	CDDP-resistance (+)	Xiao et al. [54]
EOC	LINC01554		FIGO stage, metastasis (+)	Luo et al. [55]
EOC HGSOC	MAGI2-AS3	miR-525-5p/MXD1	cell proliferation, cell cycle, migration, invasion (+)	Chang et al. [56]
EOC	MALAT1	miR-503-5p	proliferation (+) apoptosis (−)	Sun et al. [57]
HGSOC EOC	MEG3	PTEN	migration, invasion, growth, proliferation, spheroid growth in ECM (+)	Butarelli et al. [31]
		miR-30e-3p, LAMA4	angiogenesis (+)	Liu et al. [58]
		miR-376a/RASA1	proliferation (+) apoptosis (−)	Li et al. [59]
EOC	MIR503HG		proliferation, invasion (+) apoptosis (−)	Tian et al. [60]
EOC	MSC-AS1	miR-425-5p	proliferation (+) apoptosis (−)	Zhao et al. [61]
EOC	NPBWR1-2		viability, proliferation, migration, invasion (+) apoptosis (−)	Liu et al. [62]
EOC	PAXIP1-AS1		tumor grade, invasion,	Chen et al. [63]
			immune infiltration (+)	
EOC	PITPNA-AS1	miR-223-3p/RHOB	apoptosis (−)	Zhang et al. [64]
			migration, proliferation, EMT (+)	
HGSOC	PLADE	HNRNPD	proliferation, migration, invasion (−)	Liu et al. [65]
			CDDP sensitivity, apoptosis (+)	
EOC	RFPL1S-202	IFN/STAT1	CDDP, PTX cytotoxicity (+) proliferation, invasion, migration (−)	Liu et al. [66]
EOC	RP11-499E18.1	PAK2-SOX2	proliferation, migration, EMT (+)	Yang et al. [67]
EOC	SDCBP2-AS1	miR-100-5p, EPDR1	viability, migration, invasion (+) apoptosis (−)	Liu et al. [68]
EOC	SLC25A21-AS1	KCNK4, EZH2	sensitivity to PTX, CDDP (+) proliferation, invasion, migration (−)	Huang et al. [69]
		PTBP3	proliferation, metastasis (+)	Li et al. [70]
EOC	SNHG10	miR-200a-3p/BIN1	proliferation, colony formation, migration, invasion (−)	Lv et al. [71]
EOC	SNHG5	miR-23a	PTX sensitivity (+)	Lin et al. [72]
EOC	SNHG9	miR-214-5p	FIGO stage, metastasis, migration, proliferation, invasion (+)	Chen et al. [73]
EOC	TTN-AS1	miR-15b-5p/FBXW7	proliferation (+) apoptosis (−)	Miao et al. [74]
EOC	TUSC7	miR-616-5p/GSK3β	poor prognosis, proliferation, invasion, migration (+)	Zhu et al. [75]

**Table 2:** Upregulated lncRNAs in epithelial ovarian cancer. (+) increased, (−) decreased, EMT—epithelial–mesenchymal transition, PTX—paclitaxel, DTX—docetaxel, CDPP—cisplatin, CBDCA—carboplatin. All the studies included in the table were published after 2020.

Type of Ovarian Cancer	LncRNA	Target	Function	Reference
EOC	AC005224.4	miR-140-3p/SNAI2	proliferation, migration, invasion, EMT (+)	Xiong et al. [76]
EOC	ACTA2-AS1	miR-378a-3p/Wnt5a	CDDP-resistance	Lin et al. [77]
		miR-532-5p, CXCL2	proliferation and invasion (+) apoptosis (−)	Li et al. [78]
EOC	ADAMTS9-AS1	miR-587/SLC7A11	ferroptosis (+)	Cai et al. [79]
EOC	AFAP1-AS1		FIGO stage, tumor size, proliferation, metastasis	Zhou et al. [80]
		miR-107/PDK4	migration, invasion (+)	Liu et al. [81]
EOC	ARAP1-AS1	miR-4735-3p/PLAGL2	proliferation, migration, invasion (+)	Li et al. [82]
EOC	ASAP1IT1	miR-2278/LATS2	proliferation (+) apoptosis (−)	Wang et al. [83]
EOC	ASB16-AS1	miR-3918	proliferation, migration, invasion (+) apoptosis (−)	Fan et al. [84]
EOC	ATB	miR-204-3p	proliferation, invasion, migration (+) apoptosis (−)	Yuan et al. [85]
		EZH2	proliferation, invasion, migration (+)	Chen et al. [86]
		miR-204-3p/TGFβR2	viability, angiogenesis, migration (+)	Yuan et al. [87]
EOC	BBOX1-AS1	miR-361-3p/PODXL	proliferation (+) apoptosis (−)	Yao et al. [88]
EOC	BC041954	miR-197-3p; miR-23b-3p; miR-149-3pl;	FIGO stage, metastasis (+)	Lu et al. [89]
		miR-193a		
EOC	BLACAT1	miRNA-519d-3p	proliferation, migration, and invasion (+)	Yang et al. [90]
EOC	CACNA1G-AS1	FTH1-IGF2BP1	ferroptosis/ferritinophagy (−)	Jin et al. [91]
			proliferation, migration (+)	
EOC HGSOC	CASC10		apoptosis, proliferation, invasion (−)	Noriega-Rivera et al. [92]
			CDDP sensitivity (+)	
EOC OCCC	CASC15	miR-23b-3p/miR-24-3p; TGF-β/SMAD3	OS (−) migratiOn, invasion, EMT (+)	Lin et al. [93]
EOC	CCAT1	miR-454/survivin	CDDP-resistance (−)	Wang et al. [94]
EOC	CCNG1	miR-488-3p	proliferation, migration, invasion (+)	Sun et al. [95]
EOC	CDKN2A-AS1	SOSTDC1	proliferation, migration, invasion (+)	Zhao et al. [96]
		miR-143-3p/SMAD3	proliferation, invasion, migration (+) apoptosis (−)	Xu et al. [97]
EOC	CDKN2BAS	GAS6	proliferation, migration (+)	Wang et al. [98]
EOC	CRNDE	miR-423-5p/FSCN1	proliferation, invasion, migration (+)	Wang et al. [99]
		SRSF1/TIA1	CDDP-resistance (+)	Wu et al. [100]
HGSOC	CTBP1-DT	miR-188-5p/MAP3K3	proliferation, migration, invasion (+)	Liu et al. [101]
	EOC	DDUP		Ren et al. [102]
EOC	CTD-2288O8	EGFR/AKT	CDDP-resistance, viability, proliferation, invasion (+)	Liu et al. [103]
EOC	CTSLP8	PKM2/c-Myc	proliferation, CDDP-resistance, glycolysis	Li et al. [104]
		miR-199a-5p, CTSL1	autophagy, EMT (+)	Wang et al. [105]
EOC	DANCR	miR-214	viability, migration, and invasion (+)	Huang et al. [106]
EOC	DARS-AS1	miR-194-5p/RBX1	proliferation, migration, apoptosis (+)	Zhou et al. [107]
EOC	DATOC-1	miR-7	proliferation, invasion (+)	Qin et al. [108]
EOC	DLEU1	miR-429/TFAP2A	proliferation, migration, invasion (+)	Xu et al. [109]
EOC OCCC	DLX6-AS1	miR-195-5p	proliferation, migration, invasion (+) apoptosis (−)	Kong et al. [110]
EOC OCCC	DNM3	miR-193a-3p/MAP3K3	proliferation, migration, invasion (+)	He et al. [111]
EOC	DSCR8	miR-98-5p/STAT3/HIF-α	poor survival, proliferation, invasion, EMT (+) apoptosis (−)	Dong et al. [112]
EOC OCCC	DUXAP8	microRNA-29a-3p	poor prognosis, proliferation, migration	Li et al. [113]
		miR-590-5p	proliferation (+) apoptosis (−)	Meng et al. [114]
EOC	ELFN1-AS1	miR-497-3p/CLDN4	poor prognosis, proliferation, invasion, migration (+)	Jie et al. [115]
EOC	FAM83H-AS1		OS	Yuan et al. [116]
EOC	FEZF1-AS1	miR-130a-5p/SOX4	proliferation, migration, invasion (+)	Sun et al. [117]
EOC	FGD5-AS1	miR-142-5p/PD-L1	proliferation, migration, invasion (+)	Aichen et al. [118]
EOC	FGFR3-AS1		tumor grade, FIGO stage, poor prognosis, cell growth, proliferation (+) apoptosis (−)	Zhang et al. [119]
EOC	FLVCR1-AS1	miR-513/YAP1	growth, migration, invasion, and EMT (+)apoptosis (−)	Yan et al. [120]
EOC	FOXD2-AS1	miR-4492	proliferation, invasion (+)	Gao et al. [121]
EOC	FTX	miR-7515/TPD52	migration, invasion, EMT (+)	Li et al. [122]
EOC	GClnc1	p53	proliferation, migration	Li et al. [123]
		NOTCH1/NF-κB/Snail	proliferation, EMT (+)	Wu et al. [124]
EOC	H19		migration, invasion	Ma et al. [125]
		miR-29b-3p	CBDCA-resistance	Tian et al. [126]
		miRNA-140/Wnt1	proliferation, migration	Wang et al. [127]
		miR-140-5p	proliferation, invasion, migration, EMT (+)	Xu et al. [128]
EOC	HAGLROS	miRNA-26b-5p	proliferation (+) apoptosis (−)	Zhu et al. [129]
EOC	HCG11	miR-144-3p/PBX3	viability, metastasis (+)	Li et al. [130]
EOC	HCG18	miR-29a/b/TRAF4/5	proliferation, migration, EMT (+)	Zhang et al. [131]
EOC		miR-525-5p/PRC1	proliferation, invasion, migration, EMT (+)	Wang et al. [132]
HGSOC	HCP5	PTBP1	apoptosis (−) proliferation, migration (+)	Shou et al. [133]
EOC	HEIH	miR-3619-5p/CTTNBP2	poor prognosis, proliferation,	Si et al. [134]
			migration, invasion (+)	
EOC	HIF1A-AS3		proliferation (+)	Xie et al. [135]
		miR-21-3p/PEG3		Fang et al. [136]
EOC	HOTAIR	miR-222-3p/CDK19	proliferation, migration, metastasis (+)	Fan et al. [137]
		ZEB1 and TGF-β1		Zhou et al. [138]
		CHEK1		Jiang et al. [139]
		EZH2, H3K27	anoikis resistance (+)	Dai et al. [140]
		miR-138-5p, EZH2	CDDP-resistance	Zhang et al. [141]
		miR-206/TBX3	steamness of stem cells (+)	Zhang et al. [142]
EOC	HOTAIRM1		proliferation (+) apoptosis (−)	Ye et al. [143]
EOC	HOTTIP	interaction with HIF-1α	migration, invasion, viability, apoptosis	Zhang et al. [144]
		MEK/ERK	proliferation, migration (+)	Liu et al. [145]
		miR-615-3p/SMARCE1	migration, invasion (+)	
		miR-148a-3p/AKT2		Tan et al. [146]
		miR-205/ZEB2	CDDP-resistance (+)	Dong et al. [147]
EOC	HOXAAS2	miR372	metastasis. FIGO stage, proliferation (+) apoptosis (−)	Wang et al. [148]
			metastasis, proliferation, migration (+)	Eoh et al. [149]
EOC	HOXA11-AS		viability, migration, invasion (+)	Chen et al. [150]
			apoptosis, DDP sensitivity (−)	
EOC	HOXB-AS3	miR-378a-3p	proliferation, invasion, migration (+)	Xu et al. [151]
EOC	HOXC-AS3	miR-96	proliferation (+)	Yang et al. [152]
EOC HGSOC	HULC	miR-137/ITGB8	tumor growth, PTX-resistance (+)	Huang et al. [153]
EOC	IDH1-AS1	miR-518c-5p/RBM47	poor prognosis, OS, progression (+)	Zhou et al. [154]
EOC	IL21-AS1	miR-561-5p/CD24	proliferation (+) apoptosis, phagocytosis (−)	Liu et al. [155]
EOC	KCNQ1OT1	miR-142-5p/CAPN10	proliferation, metastasis (+)	Liu et al. [156]
		miR-125b-5p/CD147		Chen et al. [157]
		EIF2B5		He et al. [158]
EOC	KHDRBS3	miR17H/CLDN6	PTX sensitivity, cell proliferation, colony formation, glycolysis (+)	Wu et al. [159]
EOC	KTN1-AS1	miR-505-3p/ZNF326	proliferation, invasion, migration (+)	Xie et al. [160]
EOC	LBX2-AS1	miR-4784/KDM5C	cell proliferation, migration, stemness (+) apoptosis (−)	Gu et al. [161]
EOC	LEF1-AS1	miR-1285-3p	metastasis, FIGO stage, cell proliferation, migration, invasion (+)	Zhang et al. [162]
EOC	LINC00152	BCL6 Bcl-2, Bax Caspase-3	proliferation, invasion (+)CDDP chemonseitivity (−)	Wang et al. [163] Zou et al. [164]
EOC	LINC00176	BCL3	EMT (+)	Dai et al. [165]
EOC	LINC00273		proliferation, invasion (−)	Shu et al. [166]
EOC	LINC00452	miR-501-3p	viability, migration, invasion (+)	Yang et al. [167]
EOC	LINC00494	NFκB1, FBXO32	migration, invasion (+)	Shu et al. [168]
EOC	LINC00504	miR-1244	proliferation, apoptosis (+)	Liu et al. [169]
EOC	LINC00662	miR-375	OS (−)	Tao et al. [170]
	LINC00665	miR-148b-3p/KLF5	cell proliferation, invasion, migration (+) apoptosis (−)	Wang et al. [171]
EOC	LINC00665	miR-181a-5p/FHDC	viability, proliferation, migration (+)	Wang et al. [172]
EOC	LINC00707	miR-382-5p/LRRK2		Zhao et al. [173]
	LINC00852	miR-140-3p/AGTR1	proliferation, invasion (+)	Qiao et al. [174]
EOC	LINC00857	miR-486-5p	proliferation, migration, invasion, glycolysis (+) apoptosis (−)	Lin et al. [175]
EOC	LINC00858	miR-134-5p/RAD18	proliferation, motility, EM (+), apoptosis (−)	Xue et al. [176]
		miR-134-5p/TRIM44	proliferation, migration, invasion (+)	Li et al. [177]
EOC	LINC00909	miR-23b-3p/MRC2	proliferation, migration, invasion (+)	Yang et al. [178]
EOC	LINC00922	miR-361-3p	proliferation, migration, invasion, EMT (+)	Wang et al. [179]
EOC	LINC00958	Wnt/*β*-catenin	proliferation, migration, invasion (+)	Xie et al. [180]
EOC HGSOC OCCC	LINC01094	miR-577	FIGO stage, lymph node metastasis, cell proliferation, migration, invasion and EMT (+)	Xu et al. [181]
EOC	LINC01123	miR-516b-5p	proliferation, metastasis (+) apoptosis (−)	Dong et al. [182]
EOC	LINC01132	miRNA4315p/SOX9	proliferation, migration, invasion (+) apoptosis (−)	Zhu et al. [183]
EOC	LINC01133	miR-495-3p	migration, invasion (+)	Liu et al. [184]
EOC	LINC01215	RUNX3	proliferation, migration, invasion, EMT, metastasis (+)	Liu et al. [185]
EOC	LINC01342	miRNA-30c-2-3p	proliferation, metastasis (+)	Zhang et al. [186]
EOC	LINC01503	miR-766-5p/PD-L1	CBDCA-resistance	Li et al. [187]
EOC HGSOC	LINC01605	mut_p53	migration (+)	Coan et al. [188]
EOC	LINC01969	miR-144-5p/LARP1	migration, invasion, proliferation (+)	Chen et al. [189]
EOC	LINC02323	miR-1343-3p	proliferation, migration (+)	Li et al. [190]
EOC	LINK-A			Maleki et al. [191]
EOC	LNC00115	miR-7/ERK	CDDP-resistance, invasion, migration (+)	Jang et al. [192]
EOC	LOC102724169		cell viability, CDDP efficacy (+) apoptosis (−)	Zhou et al. [193]
EOC	LOC285194		proliferation, migration, poor prognosis (+) apoptosis (−)	Yim et al. [194]
EOC	LOC642852		invasion (+)	Filippov-Levy et al. [195]
EOC	LOC646029	miR-627-3p/SPRED1	proliferation, invasion, metastasis, poor prognosis (+)	Zhao et al. [196]
EOC	LOXL1-AS1	miR-18b-5p/VMA21	proliferation, migration, invasion, metastasis (+)	Xue et al. [197]
	LUCAT1	miR-199a-5p	proliferation, apoptosis (+)	Liu et al. [198]
EOC	MAFG-AS1	miR-339-5p	tumor stage, size, lymph node metastasis, invasion, EMT, migration (+)	
				Bai et al. [199]
EOC	MALAT1		migration, invasion (+) apoptosis (−)	Mao et al. [200]
		miRNA-22	proliferation, migration, invasion (+)	Pei et al. [201]
		miR-503-5p	proliferation (+) apoptosis (−)	Sun et al. [59]
EOC	MCF2L-AS1	IGF2BP1/IGF2/ MEK/ERK axis	PTX-resistance (+)	Zhu et al. [202]
EOC	MIAT	miR-150-5p	cell growth, migration, invasion, EMT (+)	Zhou et al. [203]
EOC	MIF-AS1	miRNA-31-5p	proliferation, migration, invasion (+)	Fan et al. [204]
EOC	MIR210HG		EMT, angiogenesis (+)	Liu et al. [205]
EOC	MIR4435-2HG	miR-128-3p/CDK14	proliferation, invasion, migration (+), apoptosis (−)	Zhu et al. [206]
EOC	MNX1-AS1	miR-4697-3p/HOXB13	CBDCA-resistance	Wu et al. [207]
		miR-744-5p/SOX12	cell proliferation, migration, invasion (+) apoptosis (−)	Shen et al. [208]
EOC	MYCN	miR-152/MKK7	poor survival, proliferation (+)	Zhang et al. [209]
EOC	MYU	miR-6827-5p	FIGO stage, metastasis, proliferation (+)	Wang et al. [210]
EOC	NCK1-AS1	miR-137	cell proliferation, migration, invasion, chemoresistance (+)	Chang et al. [211]
EOC OCC HGSOC	NEAT1	CSTF3	CDDP sensitivity (−)	Luo et al. [212]
		miR-214-3p	proliferation, migration, invasion, angiogenesis (+) apoptosis (−)	Liu et al. [213]
		miR-770-5p/PARP1	CDDP-resistance, viability (+) apoptosis (−)	Zhu et al. [214]
		miR-365	proliferation, angiogenesis	Yaun et al. [215]
		miR4500/BZW1	proliferation, colony formation, migration, invasion, glycolysis (+) apoptosis (−)	Xu et al. [216]
		let-7 g/MEST/ATGL	proliferation, migration, invasion (+)	Yin et al. [217]
		miR1321	migration, invasion (+)	Luo et al. [218]
EOC	NORAD	miR-199a-3p	proliferation, invasion, migration, EMT (+)	Xu et al. [219]
EOC	NRSN2-AS1	miR-744-5p/PRKX	migration, invasion (+)	Chen et al. [220]
		PTK2/β-catenin	proliferation, metastasis (+)	Wu et al. [221]
EOC	OIP5-AS1	miR-92a	EMT, viavbility, proliferation, migration, invasion (+) apoptosis (−)	Wang et al. [222]
		miR-324-3p/NFIB	viability, invasion, migration (+)	Liu et al. [223]
		miR-137/ZNF217	proliferation, migration, invasion, EMT (+)	Guo et al. [224]
		miR-34a	invasion, migration (+)	Jiang et al. [225]
		miR1283p/CCNG1	cell viability, migration, invasion, glycolysis (+) apoptosis (−)	Liu et al. [226]
EOC	OR3A4	KLF6	proliferation and migration (+)	Guo et al. [227]
EOC	PART1	miR-150-5p/MYB	proliferation, migration, invasion (+)	Wang et al. [228]
		miR-6884-5p/RACGAP1/RRM2	proliferation, invasion, migration (+)	Li et al. [229]
		miR-503-5p	cell viability, migration, invasion (−) apoptosis (+)	Li et al. [230]
EOC	PCA3	miR-106b-5p	cell proliferation, migration, invasion.(+) protein expression (+)	Liu et al. [231]
EOC	PLAC2	CDK2	proliferation (+)	He et al. [232]
EOC	PRLB	miR-150-5p, PRLB/RSF1	apoptosis (−) PTX-resistance (+)	Zhao et al. [233]
EOC	PRNCR1	miR-653-5p/ELF2	invasion, migration, proliferation (+)	Qi et al. [234]
EOC	PRPF6	SNHG16-L/CEBPB/GATA3	FIGO stage, metastasis, PTX-resistance progression (+)	Wang et al. [235]
EOC	PSMA3-AS1	miR-378a-3p/GALNT3	proliferation, migration, invasion (+)	Xu et al. [236]
EOC	PTAL	miR-101/fn1	invasion, migration (+)	Liang et al. [237]
EOC	PURPL	miR-338-3p	FIGO stage, metastasis (+)	Zhang et al. [238]
EOC OCCC	PVT1	CTGF	proliferation, migration, invasion, EMT (+)	Dong et al. [239]
		miR-148a/AGO1	FIGO stage, poor survival rate, metastasis, cell viability, proliferation (+) apoptosis (−)	Wu et al. [240]
		miR-149-5p/FOXM1	proliferation, migration, invasion (+)	Li et al. [241]
		miR-370, miR-576, FOXM1	migration, invasion, proliferation,	Yi et al. [242]
			chemoresistance (+)	
		miR-543/SERPINI1	proliferation, migration, invasion (+) apoptosis (−)	Qu et al. [243]
			cell survival, growth, migration, chemoresistance (+)	Tabury et al. [244]
EOC	RAD51-AS1	miR-140-3p/EIF5A2	poor prognosis (+)	Zhao et al. [245]
EOC	RBAT1		FIGO stage, metastasis (+)	Luo et al. [246]
EOC	RHPN1-AS1	PI3K/AKT	viability, migration, invasion, tumor growth (+)	Cui et al. [247]
		miR-1299	proliferation, migration, invasion, low survival rate (+)	Zhao et al. [248]
		miR-665/Akt3	cell proliferation, migration, invasion (+)	Zhao et al. [249]
		miR-596/LETM1	proliferation, invasion, viability (+)	Wang et al. [250]
		miR-6884-5p	proliferation, viability, adhesion, migration (+) apoptosis (−)	Cui et al. [251]
		miR4855p	proliferation, apoptosis, adhesion, migration (+)	Cui et al. [252]
EOC HGSOC	RMRP	miR-580-3p	proliferation (+) apoptosis (−) PTX-resistance (+)	Li et al. [253]
EOC	RP5	miR-545-5p/PTP4A1	proliferation, metastasis (+)	Sun et al. [254]
EOC	SBF2-AS1	miR-338-3p	proliferation, invasion (+)	Luan et al. [255]
EOC	SCAMP1	miR-137/CXCL12	invasion, angiogenesis (+)	Song et al. [256]
EOC	SDHAP1	miR-4465/EIF4G2	PTX-resistance (+)	Zhao et al. [257]
EOC OCCC	SNHG1	miR-454/ZEB1	proliferation (+)	Wu et al. [258]
EOC HGSOC	SNHG12		CBDCA sensitivity (+)	Abildgaard et al. [259]
EOC OCCC	SNHG17	miR-18a-5p	migration and invasion (+)	Zhang et al. [260]
		miR-370-3p/CDK6	poor prognosis, migration, proliferation (+)	Wang et al. [261]
		CCL13-CCR2	proliferation, invasion, migration, EMT, metastasis (+)	Liang et al. [262]
		miR-214-3p	poor prognosis, proliferation (+) apoptosis (−)	Pan et al. [263]
		miR-485-5p/AKT1	proliferation (+) apoptosis (−)	Wang et al. [264]
EOC	SNHG20	miR-148a/ROCK1	invasion, migration, proliferation (+) apoptosis (−)	Yang et al. [265]
		miR-217	cell proliferation, invasion (+)	Xing et al. [266]
		miR-338-3p/MCL1	proliferation, migration, invasion, EMT (+) apoptosis (−)	Wang et al. [267]
EOC	SNHG22	SP1	poor prognosis, glycolysis, proliferation (+)	Guan et al. [268]
EOC	SNHG25	COMP	proliferation, migration, invasion (+) apoptosis (−)	Liu et al. [269]
EOC	SNHG3	miR-139-5p	proliferation, migration (+)	Zhang et al. [270]
HGSOC		miR-339-5p/TRPC3	poor prognosis, apoptosis (+)	Liu et al. [271]
EOC	SNHG4	miR-98-5p/TMED5	proliferation, migration, invasion (+) apoptosis (−)	Liu et al. [272]
EOC	SNHG6	miR-543/YAP1	proliferation, migration, invasion, EMT	Su et al. [273]
EOC HGSOC OCCC	SHNG7	EIF4G2	cell viability, migration, invasion, PTX-resistance (+)	Zhang et al. [274]
		KLF2, EZH2	invasion and proliferation (+)	Bai et al. [275]
EOC	SNHG8	Wnt/β-catenin	proliferation, migration, EMT (+)	Miao et al. [276]
EOC HGSOC	SPOCD1-AS	G3BP1		Wang et al. [277]
EOC	SRA		proliferation, invasion, migration (+)	Kim et al. [278]
EOC	TC0101441	KiSS1	invasion, migration, metastasis, EMT (+)	Qiu et al. [279]
EOC HGSOC	THOR	IL-6/STAT3	poor prognosis, proliferation, invasion (+)	Ge et al. [280]
EOC HGSOC	THUMPD3-AS1	miR-320d/ARF1	apoptosis (−) viability (+)	Mu et al. [281]
EOC	TLR8-AS1		metastasis, chemoresistance (+)	Xu et al. [282]
EOC	TMPO-AS1	LCN2	migration, proliferation, invasion, angiogenesis (+)	Zhao et al. [283]
		miR-200c/TMEFF2	invasion, migration, and 5-Fu resistance, EMT (+)	Li et al. [284]
EOC	TONSL-AS1	miR-490-3p/CDK1	proliferation (+)	Liu et al. [285]
EOC HGSOC	TRPM2-AS1	miR-138-5p/*SDC3*	proliferation, invasion, migration, PTX-resistance (+) apoptosis (−)	Ding et al. [286]
EOC	TUG1	miR-582-3p	viability and migration (+)	Dai et al. [287]
		miR-29b-3p	PTX-resistance (+)	Gu et al. [288]
		miR-1299/NOTCH3	proliferation (+)	Pei et al. [289]
		miR-186-5p/ZEB1	proliferation, invasion (+)	Zhan et al. [290]
		miR-4687-3p, miR-6088	CDDP sensitivity (+)	Sonobe et al. [291]
EOC	UCA1	miR-27a-5p/UBE2N	CDDP-resistance	Wambecke et al. [292]
EOC HGSOC		miR-654-5p/SIK2	proliferation, migration, invasion, PTX-resistance (+) apoptosis (−)	Li et al. [293]
EOC	UNC5B-AS1	H3K27me, NDRG2	proliferation (+) apoptosis (−)	Wang et al. [294]
EOC OCCC	USP2-AS1	miR-520d-3p	proliferation, migration (+)	Guo et al. [295]
EOC	USP30-AS1			Xiong et al. [296]
EOC	WDFY3-AS2	miR-139-5p/SDC4	CDDP-resistance, cell proliferation, migration, invasion (+) apoptosis (−)	Wu et al. [297]
EOC	XIST	miR-506-3p/FOXP	apoptosis (−) CBDCA -resistance, autophagy (+)	Xia et al. [298]
OCCC		miR-335/BCL2L2	proliferation invasion, migration (+)	Meng et al. [299]
		miR-93-5p/KMT2C	PTX resistance (+)	Huang et al. [300]
		miR-149-3p	poor prognosis, proliferation, invasion, migration (+) apoptosis (−)	Jiang et al. [301]
EOC	ZEB1-AS1	MMP19	PTX, CDDP-resistance (−)	Dai et al. [302]

## Data Availability

Data supporting the findings of this study are available from the corresponding author upon reasonable request.

## References

[1] Wang J.Y., Lu A.Q., Chen L.J. (2019). LncRNAs in ovarian cancer. Clin. Chim. Acta.

[2] Rusu I., Pirlog R., Chiroi P., Nutu A., Puia V.R., Fetti A.C., Rusu D.R., Berindan-Neagoe I., Al Hajjar N. (2022). The Implications of Noncoding RNAs in the Evolution and Progression of Nonalcoholic Fatty Liver Disease (NAFLD)-Related, HCC. Int. J. Mol. Sci..

[3] Sun R., Qin C., Jiang B., Fang S., Pan X., Peng L., Liu Z., Li W., Li Y., Li G. (2016). Down-regulation of MALAT1 inhibits cervical cancer cell invasion and metastasis by inhibition of epithelial-mesenchymal transition. Mol. Biosyst..

[4] Du Y., Geng G., Zhao C., Gao T., Wei B. (2022). LncRNA MEG3 promotes cisplatin sensitivity of cervical cancer cells by regulating the miR-21/PTEN axis. BMC Cancer.

[5] Zhang J., Yao T., Wang Y., Yu J., Liu Y., Lin Z. (2016). Long noncoding RNA MEG3 is downregulated in cervical cancer and affects cell proliferation and apoptosis by regulating miR-21. Cancer Biol. Ther..

[6] Huang J., Ke P., Guo L., Wang W., Tan H., Liang Y., Yao S. (2014). Lentivirus-mediated RNA interference targeting the long noncoding RNA HOTAIR inhibits proliferation and invasion of endometrial carcinoma cells in vitro and in vivo. Int. J. Gynecol. Cancer.

[7] Guo C., Song W.Q., Sun P., Jin L., Dai H.Y. (2015). LncRNA-GAS5 induces PTEN expression through inhibiting miR-103 in endometrial cancer cells. J. Biomed. Sci..

[8] Jiang J., Duan M., Wang Z., Lai Y., Zhang C., Duan C. (2024). RNA epigenetics in pulmonary diseases: Insights into methylation modification of lncRNAs in lung cancer. Biomed. Pharmacother..

[9] Dai S., Wang Q., Lyu Y., Chen Z., Liu X., Zhao G., Zhang H. (2024). LncRNA AC100826.1 regulated PLCB1 to promote progression in non-small cell lung cancer. Thorac. Cancer.

[10] Zhu W., Huang H., Hu Z., Gu Y., Zhang R., Shu H., Liu H., Sun X. (2024). Comprehensive Transcriptome Analysis Expands lncRNA Functional Profiles in Breast Cancer. Int. J. Mol. Sci..

[11] Thangavelu L., Moglad E., Gupta G., Menon S.V., Gaur A., Sharma S., Kaur M., Chahar M., Sivaprasad G.V., Deorari M. (2024). GAS5 lncRNA: A biomarker and therapeutic target in breast cancer. Pathol. Res. Pract..

[12] Chen Y., Zhou Y., Chen J., Yang J., Yuan Y., Wu W. (2024). Exosomal lncRNA SNHG12 promotes angiogenesis and breast cancer progression. Breast Cancer.

[13] Li X., Meng Y., Gu B. (2024). A novel immune-related lncRNA as a prognostic biomarker in HER2+ breast cancer. Oncol. Lett..

[14] Yu J.M., Sun C.Q., Xu H.H., Jiang Y.L., Jiang X.Y., Ni S.Q., Zhao T.Y., Liu L.X. (2024). Navigating the labyrinth of long non-coding RNAs in colorectal cancer: From chemoresistance to autophagy. World J. Gastrointest. Oncol..

[15] Chen T., Jiang Q., Wang Z., Zhang H., Fu Z. (2024). The roles of lncRNA AP001469.3 in clinical implications, immune landscape and carcinogenesis of colorectal cancer. Transl. Cancer Res..

[16] Luo X.J., Lu Y.X., Wang Y., Huang R., Liu J., Jin Y., Liu Z.K., Liu Z.X., Huang Q.T., Pu H.Y. (2024). M6A-modified lncRNA FAM83H-AS1 promotes colorectal cancer progression through PTBP1. Cancer Lett..

[17] Mao R., Xu C., Zhang Q., Wang Z., Liu Y., Peng Y., Li M. (2024). Predictive significance of glycolysis-associated lncRNA profiles in colorectal cancer progression. BMC Med. Genom..

[18] Lheureux S., Braunstein M., Oza A.M. (2019). Epithelial ovarian cancer: Evolution of management in the era of precision medicine. CA Cancer J. Clin..

[19] Kuroki L., Guntupalli S.R. (2020). Treatment of epithelial ovarian cancer. BMJ.

[20] Konstantinopoulos P.A., Norquist B., Lacchetti C., Armstrong D., Grisham R.N., Goodfellow P.J., Kohn E.C., Levine D.A., Liu J.F., Lu K.H. (2020). Germline and Somatic Tumor Testing in Epithelial Ovarian Cancer: ASCO Guideline. J. Clin. Oncol..

[21] Kaku T., Ogawa S., Kawano Y., Ohishi Y., Kobayashi H., Hirakawa T., Nakano H. (2003). Histological classification of ovarian cancer. Med. Electron. Microsc..

[22] Mok S.C., Kwong J., Welch W.R., Samimi G., Ozbun L., Bonome T., Birrer M.J., Berkowitz R.S., Wong K.K. (2007). Etiology and pathogenesis of epithelial ovarian cancer. Dis. Markers.

[23] Zhang R., Siu M.K.Y., Ngan H.Y.S., Chan K.K.L. (2022). Molecular Biomarkers for the Early Detection of Ovarian Cancer. Int. J. Mol. Sci..

[24] Chien J.R., Aletti G., Bell D.A., Keeney G.L., Shridhar V., Hartmann L.C. (2007). Molecular pathogenesis and therapeutic targets in epithelial ovarian cancer. J. Cell Biochem..

[25] Pejovic T., Thisted S., White M., Nezhat F.R. (2020). Endometriosis and Endometriosis-Associated Ovarian Cancer (EAOC). Adv. Exp. Med. Biol..

[26] Kok V.C., Tsai H.J., Su C.F., Lee C.K. (2015). The risks for ovarian, endometrial, breast, colorectal, and other cancers in women with newly diagnosed endometriosis or adenomyosis: A population-based study. Int. J. Gynecol. Cancer..

[27] Stewart C.J., Leung Y., Walsh M.D., Walters R.J., Young J.P., Buchanan D.D. (2012). KRAS mutations in ovarian low-grade endometrioid adenocarcinoma: Association with concurrent endometriosis. Hum. Pathol..

[28] Zhang J., Yang Z.M., Huang Y., Wang K.N., Xie Y., Yang N. (2021). LncRNA GAS5 inhibits the proliferation and invasion of ovarian clear cell carcinoma via the miR-31-5p/ARID1A axis. Kaohsiung J. Med. Sci..

[29] Wu Y., Deng Y., Guo Q., Zhu J., Cao L., Guo X., Xu F., Weng W., Ju X., Wu X. (2019). Long non-coding RNA SNHG6 promotes cell proliferation and migration through sponging miR-4465 in ovarian clear cell carcinoma. J. Cell Mol. Med..

[30] Rethlefsen M.L., Kirtley S., Waffenschmidt S., Ayala A.P., Moher D., Page M.J., Koffel J.B., PRISMA-S Group (2021). PRISMA-S: An extension to the PRISMA Statement for Reporting Literature Searches in Systematic Reviews. Syst. Rev..

[31] Buttarelli M., De Donato M., Raspaglio G., Babini G., Ciucci A., Martinelli E., Baccaro P., Pasciuto T., Fagotti A., Scambia G. (2020). Clinical Value of lncRNA MEG3 in High-Grade Serous Ovarian Cancer. Cancers.

[32] Filippov-Levy N., Cohen-Schussheim H., Tropé C.G., Hetland Falkenthal T.E., Smith Y., Davidson B., Reich R. (2018). Expression and clinical role of long non-coding RNA in high-grade serous carcinoma. Gynecol. Oncol..

[33] Lin X., Tang X., Zheng T., Qiu J., Hua K. (2020). Long non-coding RNA AOC4P suppresses epithelial ovarian cancer metastasis by regulating epithelial-mesenchymal transition. J. Ovarian Res..

[34] Xu H., Tang Y., Liu L., Yan J., Qin L. (2022). Downregulation of lncRNA ASMTL-AS1 in Epithelial Ovarian Cancer Correlates with Worse Prognosis and Cancer Progression. Horm. Metab. Res..

[35] Cui K., Zhu G. (2020). LncRNA CTBP1-AS2 regulates miR-216a/PTEN to suppress ovarian cancer cell proliferation. J. Ovarian Res..

[36] Sun T., Yang P., Gao Y. (2020). Long non-coding RNA EPB41L4A-AS2 suppresses progression of ovarian cancer by sequestering microRNA-103a to upregulate transcription factor RUNX1T1. Exp. Physiol..

[37] Yao C., Zeng L., Liu Q., Qiu X., Chen C. (2023). LncRNA FAM225B Regulates PDIA4-Mediated Ovarian Cancer Cell Invasion and Migration via Modulating Transcription Factor DDX17. Breast J..

[38] Zhang W., Shi J., Liu G. (2023). Abnormal expression of long non-coding RNA FGD5-AS1 affects the development of ovarian cancer through regulating miR-107/RBBP6 axis. Chin. J. Physiol..

[39] Dong Q., Long X., Cheng J., Wang W., Tian Q., Di W. (2021). LncRNA GAS5 suppresses ovarian cancer progression by targeting the miR-96-5p/PTEN axis. Ann. Transl. Med..

[40] Zhou L., Jiang H., Lin L., Li Y., Li J. (2023). lncRNA GAS5 suppression of the malignant phenotype of ovarian cancer via the miR-23a-WT1 axis. Ann. Transl. Med..

[41] Zhang T., Leng Y., Duan M., Li Z., Ma Y., Huang C., Shi Q., Wang Y., Wang C., Liu D. (2023). LncRNA GAS5-hnRNPK axis inhibited ovarian cancer progression via inhibition of AKT signaling in ovarian cancer cells. Discov. Oncol..

[42] Fang Y.J., Jiang P., Zhai H., Dong J.S. (2020). LncRNA GAS8-AS1 Inhibits Ovarian Cancer Progression Through Activating Beclin1-Mediated Autophagy. Onco Targets Ther..

[43] Gokulnath P., de Cristofaro T., Manipur I., Di Palma T., Soriano A.A., Guarracino M.R., Zannini M. (2020). Long Non-Coding RNA HAND2-AS1 Acts as a Tumor Suppressor in High-Grade Serous Ovarian Carcinoma. Int. J. Mol. Sci..

[44] Li L., Li L., Hu L., Li T., Xie D., Liu X. (2021). Long non-coding RNA HAND2-AS1/miR-106a/PTEN axis re-sensitizes cisplatin-resistant ovarian cells to cisplatin treatment. Mol. Med. Rep..

[45] Chen X., Yang Y., Sun J., Hu C., Ge X., Li R. (2022). LncRNA HCG11 represses ovarian cancer cell growth via AKT signaling pathway. J. Obstet. Gynaecol. Res..

[46] Chao H., Zhang M., Hou H., Zhang Z., Li N. (2020). HOTAIRM1 suppresses cell proliferation and invasion in ovarian cancer through facilitating ARHGAP24 expression by sponging miR-106a-5p. Life Sci..

[47] Yang X., Zhou S., Li C., Huang L., Chen C., Tang X., Su X., Zhu H. (2022). Downregulation of LEMD1-AS1 and Its Influences on the Diagnosis, Prognosis, and Immune Infiltrates of Epithelial Ovarian Cancer. Dis. Markers.

[48] Guo R., Qin Y. (2020). LEMD1-AS1 Suppresses Ovarian Cancer Progression Through Regulating miR-183-5p/TP53 Axis. Onco Targets Ther..

[49] Liu F., Cao L., Zhang Y., Xia X., Ji Y. (2022). LncRNA LIFR-AS1 overexpression suppressed the progression of serous ovarian carcinoma. J. Clin. Lab. Anal..

[50] Hao T., Huang S., Han F. (2020). LINC-PINT suppresses tumour cell proliferation, migration and invasion through targeting miR-374a-5p in ovarian cancer. Cell Biochem. Funct..

[51] Liu J., Zhu Y., Wang H., Han C., Wang Y., Tang R. (2024). LINC00629, a HOXB4-downregulated long noncoding RNA, inhibits glycolysis and ovarian cancer progression by destabilizing c-Myc. Cancer Sci..

[52] Shu C., Wang W., Wu L., Qi C., Yan W., Lu W., Tian J., Shang A. (2023). LINC00936/microRNA-221-3p Regulates Tumor Progression in Ovarian Cancer by Interacting with LAMA3. Recent Patents Anti-Cancer Drug Discov..

[53] Xu H., Mao H.L., Zhao X.R., Li Y., Liu P.S. (2020). MiR-29c-3p, a target miRNA of LINC01296, accelerates tumor malignancy: Therapeutic potential of a LINC01296/miR-29c-3p axis in ovarian cancer. J. Ovarian Res..

[54] Xiao L., Shi X.Y., Li Z.L., Li M., Zhang M.M., Yan S.J., Wei Z.L. (2021). Downregulation of LINC01508 contributes to cisplatin resistance in ovarian cancer via the regulation of the Hippo-YAP pathway. J. Gynecol. Oncol..

[55] Luo T., Jiang Y., Yang J. (2021). Long Noncoding RNA LINC01554 as a Novel Biomarker for Diagnosis and Prognosis Prediction of Epithelial Ovarian Cancer. Dis. Markers.

[56] Chang H., Zhang X., Li B., Meng X. (2020). MAGI2-AS3 suppresses MYC signaling to inhibit cell proliferation and migration in ovarian cancer through targeting miR-525-5p/MXD1 axis. Cancer Med..

[57] Sun Q., Li Q., Xie F. (2019). LncRNA-MALAT1 regulates proliferation and apoptosis of ovarian cancer cells by targeting miR-503-5p. Onco Targets Ther..

[58] Liu Y., Xu Y., Ding L., Yu L., Zhang B., Wei D. (2020). LncRNA MEG3 suppressed the progression of ovarian cancer via sponging miR-30e-3p and regulating LAMA4 expression. Cancer Cell Int..

[59] Li Y., Zhang L., Zhao Y., Peng H., Zhang N., Bai W. (2023). MEG3 sponges miRNA-376a and YBX1 to regulate angiogenesis in ovarian cancer endothelial cells. Heliyon.

[60] Tian J., Yang L., Wang Z., Yan H. (2022). MIR503HG impeded ovarian cancer progression by interacting with SPI1 and preventing TMEFF1 transcription. Aging.

[61] Zhao Y., Yuan D., Zhu D., Xu T., Huang A., Jiang L., Liu C., Qian H., Bu X. (2021). LncRNA-MSC-AS1 inhibits the ovarian cancer progression by targeting miR-425-5p. J. Ovarian Res..

[62] Liu S., Du Q., Rao Y., Liu C., Qu P. (2020). Long non-coding RNA NPBWR1-2 affects the development of ovarian cancer via multiple microRNAs. Oncol. Lett..

[63] Chen B., Lu X., Zhou Q., Chen Q., Zhu S., Li G., Liu H. (2023). PAXIP1-AS1 is associated with immune infiltration and predicts poor prognosis in ovarian cancer. PLoS ONE.

[64] Zhang F., Zhang M., Chen Z., Yu B., He X., Luo Y., Ai F., Hu W. (2024). PITPNA-AS1 Inhibits Cell Proliferation and Migration in Ovarian Cancer by Regulating the MIR-223-3p/RHOB Axis. Rev. Investig. Clin..

[65] Liu H., Deng S., Yao X., Liu Y., Qian L., Wang Y., Zhang T., Shan G., Chen L., Zhou Y. (2024). Ascites exosomal lncRNA PLADE enhances platinum sensitivity by inducing R-loops in ovarian cancer. Oncogene.

[66] Liu S., Chen X., Huang K., Xiong X., Shi Y., Wang X., Pan X., Cong Y., Sun Y., Ge L. (2023). Long noncoding RNA RFPL1S-202 inhibits ovarian cancer progression by downregulating the IFN-β/STAT1 signaling. Exp. Cell Res..

[67] Yang J., Peng S., Zhang K. (2021). LncRNA RP11-499E18.1 Inhibits Proliferation, Migration, and Epithelial-Mesenchymal Transition Process of Ovarian Cancer Cells by Dissociating PAK2-SOX2 Interaction. Front. Cell Dev. Biol..

[68] Liu X., Liu C., Zhang A., Wang Q., Ge J., Li Q., Xiao J. (2021). Long non-coding RNA SDCBP2-AS1 delays the progression of ovarian cancer via microRNA-100-5p-targeted EPDR1. World J. Surg. Oncol..

[69] Huang K., Chen X., Geng Z., Xiong X., Cong Y., Pan X., Liu S., Ge L., Xu J., Jia X. (2023). LncRNA SLC25A21-AS1 increases the chemosensitivity and inhibits the progression of ovarian cancer by upregulating the expression of KCNK4. Funct. Integr. Genom..

[70] Li S., Shen S., Ge W., Cen Y., Zhang S., Cheng X., Wang X., Xie X., Lu W. (2023). Long non-coding RNA SLC25A21-AS1 inhibits the development of epithelial ovarian cancer by specifically inducing PTBP3 degradation. Biomark. Res..

[71] Lv W., Jia Y., Wang J., Duan Y., Wang X., Liu T., Hao S., Liu L. (2022). Long non-coding RNA SNHG10 upregulates BIN1 to suppress the tumorigenesis and epithelial-mesenchymal transition of epithelial ovarian cancer via sponging miR-200a-3p. Cell Death Discov..

[72] Lin H., Shen L., Lin Q., Dong C., Maswela B., Illahi G.S., Wu X. (2020). SNHG5 enhances Paclitaxel sensitivity of ovarian cancer cells through sponging miR-23a. Biomed. Pharmacother..

[73] Chen G.Y., Zhang Z.S., Chen Y., Li Y. (2021). Long non-coding RNA SNHG9 inhibits ovarian cancer progression by sponging microRNA-214-5p. Oncol. Lett..

[74] Miao S., Wang J., Xuan L., Liu X. (2020). LncRNA TTN-AS1 acts as sponge for miR-15b-5p to regulate FBXW7 expression in ovarian cancer. Biofactors.

[75] Zhu L.M., Li N. (2020). Downregulation of long noncoding RNA TUSC7 promoted cell growth, invasion and migration through sponging with miR-616-5p/GSK3β pathway in ovarian cancer. Eur. Rev. Med. Pharmacol. Sci..

[76] Xiong T., Wang Y., Zhang Y., Yuan J., Zhu C., Jiang W. (2023). lncRNA AC005224.4/miR-140-3p/SNAI2 regulating axis facilitates the invasion and metastasis of ovarian cancer through epithelial-mesenchymal transition. Chin. Med. J..

[77] Lin C., Zheng M., Yang Y., Chen Y., Zhang X., Zhu L., Zhang H. (2022). Knockdown of lncRNA ACTA2-AS1 reverses cisplatin resistance of ovarian cancer cells via inhibition of miR-378a-3p-regulated Wnt5a. Bioengineered.

[78] Li Y., Yang Z., Chen J. (2021). Mechanism underlying the regulation of lncRNA ACTA2-AS1 on CXCL2 by absorbing miRNA-532-5p as ceRNA in the development of ovarian cancer. Int. J. Clin. Exp. Pathol..

[79] Cai L., Hu X., Ye L., Bai P., Jie Y., Shu K. (2022). Long non-coding RNA ADAMTS9-AS1 attenuates ferroptosis by Targeting microRNA-587/solute carrier family 7 member 11 axis in epithelial ovarian cancer. Bioengineered.

[80] Zhou F., Xu X., Wei W., Chen X., Sun L. (2023). Regulations of Exosomal-Transmitted AFAP1-AS1 LncRNA on Ovarian Cancer Cell Migration and Invasion. Discov. Med..

[81] Liu B., Yan L., Chi Y., Sun Y., Yang X. (2021). Long non-coding RNA AFAP1-AS1 facilitates ovarian cancer progression by regulating the miR-107/PDK4 axis. J. Ovarian Res..

[82] Li C., Dong B., Xu X., Li Y., Wang Y., Li X. (2022). Correction to: LncRNA ARAP1-AS1 aggravates the malignant phenotypes of ovarian cancer cells through sponging miR-4735-3p to enhance PLAGL2 expression. Cytotechnology.

[83] Wang K., Hu Y.B., Zhao Y., Ye C. (2021). Long non-coding RNA ASAP1-IT1 suppresses ovarian cancer progression by regulating Hippo/YAP signaling. Int. J. Mol. Med..

[84] Fan Y., Wang L., Han X., Ma H., Zhang N., She L. (2023). LncRNA ASB16-AS1 accelerates cellular process and chemoresistance of ovarian cancer cells by regulating GOLM1 expression via targeting miR-3918. Biochem. Biophys. Res. Commun..

[85] Yuan D., Zhang X., Zhao Y., Qian H., Wang H., He C., Liu X., Guo T., Lin M., Yu H. (2020). Role of lncRNA-ATB in ovarian cancer and its mechanisms of action. Exp. Ther. Med..

[86] Chen X.J., An N. (2021). Long noncoding RNA ATB promotes ovarian cancer tumorigenesis by mediating histone H3 lysine 27 trimethylation through binding to EZH2. J. Cell Mol. Med..

[87] Yuan D., Guo T., Zhu D., Ge H., Zhao Y., Huang A., Wang X., Cao X., He C., Qian H. (2022). Exosomal lncRNA ATB Derived from Ovarian Cancer Cells Promotes Angiogenesis via Regulating miR-204-3p/TGFβR2 Axis. Cancer Manag. Res..

[88] Yao H., Chen R., Yang Y., Jiang J. (2021). LncRNA BBOX1-AS1 Aggravates the Development of Ovarian Cancer by Sequestering miR-361-3p to Augment PODXL Expression. Reprod. Sci..

[89] Lu Y.M., Guo Y.R., Zhou M.Y., Wang Y. (2022). Expression and clinical significance of lncRNA BC041954 in ovarian cancer. Exp. Ther. Med..

[90] Yang H., Qi Y., Wang X.L., Gu J.J., Shi T.M. (2020). Down-regulation of lncRNA BLACAT1 inhibits ovarian cancer progression by suppressing the Wnt/β-catenin signaling pathway via regulating miR-519d-3p. Mol. Cell Biochem..

[91] Jin Y., Qiu J., Lu X., Ma Y., Li G. (2023). LncRNA CACNA1G-AS1 up-regulates FTH1 to inhibit ferroptosis and promote malignant phenotypes in ovarian cancer cells. Oncol. Res..

[92] Noriega-Rivera R., Rivera-Serrano M., Rabelo-Fernandez R.J., Pérez-Santiago J., Valiyeva F., Vivas-Mejía P.E. (2022). Upregulation of the Long Noncoding RNA CASC10 Promotes Cisplatin Resistance in High-Grade Serous Ovarian Cancer. Int. J. Mol. Sci..

[93] Lin H., Xu X., Chen K., Fu Z., Wang S., Chen Y., Zhang H., Niu Y., Chen H., Yu H. (2022). LncRNA CASC15, MiR-23b Cluster and SMAD3 form a Novel Positive Feedback Loop to promote Epithelial-Mesenchymal Transition and Metastasis in Ovarian Cancer. Int. J. Biol. Sci..

[94] Wang D.Y., Li N., Cui Y.L. (2020). Long Non-coding RNA CCAT1 Sponges miR-454 to Promote Chemoresistance of Ovarian Cancer Cells to Cisplatin by Regulation of Surviving. Cancer Res. Treat..

[95] Sun M., Chen Y., Liu X., Cui Y. (2021). LncRNACASC9 promotes proliferation, metastasis, and cell cycle inovarian carcinoma cells through cyclinG1/TP53/MMP7 signaling. Bioengineered.

[96] Zhao Q., Dong D., Chu H., Man L., Huang X., Yin L., Zhao D., Mu L., Gao C., Che J. (2021). lncRNA CDKN2A-AS1 facilitates tumorigenesis and progression of epithelial ovarian cancer via modulating the SOSTDC1-mediated BMP-SMAD signaling pathway. Cell Cycle.

[97] Xu C., Zhai J., Fu Y. (2020). LncRNA CDKN2B-AS1 promotes the progression of ovarian cancer by miR-143-3p/SMAD3 axis and predicts a poor prognosis. Neoplasma.

[98] Wang H.M., Shen S.L., Li N.M., Su H.F., Li W.Y. (2020). LncRNA CDKN2BAS aggravates the progression of ovarian cancer by positively interacting with GAS6. Eur. Rev. Med. Pharmacol. Sci..

[99] Wang Q., Wang L.X., Zhang C.Y., Bai N., Feng C., Zhang Z.M., Wang L., Gao Z.Z. (2022). LncRNA CRNDE promotes cell proliferation, migration and invasion of ovarian cancer via miR-423-5p/FSCN1 axis. Mol. Cell Biochem..

[100] Wu J., Ni X., Yu Z., Wu S., Liu Z. (2022). CRNDE inducing cisplatin resistance through SRSF1/TIA1 signaling pathway in ovarian cancer. Pathol. Res. Pract..

[101] Liu P., Fu R., Chen K., Zhang L., Wang S., Liang W., Zou H., Tao L., Jia W. (2021). ETV5-mediated upregulation of lncRNA CTBP1-DT as a ceRNA facilitates HGSOC progression by regulating miR-188-5p/MAP3K3 axis. Cell Death Dis..

[102] Ren L., Qing X., Wei J., Mo H., Liu Y., Zhi Y., Lu W., Zheng M., Zhang W., Chen Y. (2023). The DDUP protein encoded by the DNA damage-induced CTBP1-DT lncRNA confers cisplatin resistance in ovarian cancer. Cell Death Dis..

[103] Liu T., Shen J., He Q., Xu S. (2022). Identification of a Novel Immune-Related lncRNA CTD-2288O8.1 Regulating Cisplatin Resistance in Ovarian Cancer Based on Integrated Analysis. Front. Genet..

[104] Li X., Zhang Y., Wang X., Lin F., Cheng X., Wang Z., Wang X. (2022). Long non-coding RNA CTSLP8 mediates ovarian cancer progression and chemotherapy resistance by modulating cellular glycolysis and regulating c-Myc expression through PKM2. Cell Biol. Toxicol..

[105] Wang X., Li X., Lin F., Sun H., Lin Y., Wang Z., Wang X. (2021). The lnc-CTSLP8 upregulates CTSL1 as a competitive endogenous RNA and promotes ovarian cancer metastasis. J. Exp. Clin. Cancer Res..

[106] Huang P., Qi B., Yao H., Zhang L., Li Y., Li Q. (2020). Knockdown of DANCR Suppressed the Biological Behaviors of Ovarian Cancer Cells Treated with Transforming Growth Factor-β (TGF-β) by Sponging MiR-214. Med. Sci. Monit..

[107] Zhou M., Cheng H., Fu Y., Zhang J. (2021). Long noncoding RNA DARS-AS1 regulates TP53 ubiquitination and affects ovarian cancer progression by modulation miR-194-5p/RBX1 axis. J. Biochem. Mol. Toxicol..

[108] Qin W., Miao Y., Sun G., Chen S., Zang Y.S., Dong C. (2021). Long noncoding RNA DATOC-1 that associate with DICER promotes development in epithelial ovarian cancer by upregulating miR-7 expression. Transl. Cancer Res..

[109] Xu H., Wang L., Jiang X. (2021). Silencing of lncRNA DLEU1 inhibits tumorigenesis of ovarian cancer via regulating miR-429/TFAP2A axis. Mol. Cell Biochem..

[110] Kong L., Zhang C. (2020). LncRNA DLX6-AS1 aggravates the development of ovarian cancer via modulating FHL2 by sponging miR-195-5p. Cancer Cell Int..

[111] He L., He G. (2021). DNM3OS Facilitates Ovarian Cancer Progression by Regulating miR-193a-3p/MAP3K3 Axis. Yonsei Med. J..

[112] Dong L., Cao X., Luo Y., Zhang G., Zhang D. (2020). A Positive Feedback Loop of lncRNA DSCR8/miR-98-5p/STAT3/HIF-1α Plays a Role in the Progression of Ovarian Cancer. Front. Oncol..

[113] Li L.M., Hao S.J., Ni M., Jin S., Tian Y.Q. (2021). DUXAP8 promotes the proliferation and migration of ovarian cancer cells via down-regulating microRNA-29a-3p expression. Eur. Rev. Med. Pharmacol. Sci..

[114] Meng Q., Li Z., Pan J., Sun X. (2020). Long noncoding RNA DUXAP8 regulates proliferation and apoptosis of ovarian cancer cells via targeting miR-590-5p. Hum. Cell.

[115] Jie Y., Ye L., Chen H., Yu X., Cai L., He W., Fu Y. (2020). ELFN1-AS1 accelerates cell proliferation, invasion and migration via regulating miR-497-3p/CLDN4 axis in ovarian cancer. Bioengineered.

[116] Yuan X., Huang Y., Guo M., Hu X., Li P. (2021). Long non-coding RNA FAM83H-AS1 acts as a potential oncogenic driver in human ovarian cancer. J. Ovarian Res..

[117] Sun Z., Gao S., Xuan L., Liu X. (2020). Long non-coding RNA FEZF1-AS1 induced progression of ovarian cancer via regulating miR-130a-5p/SOX4 axis. J. Cell Mol. Med..

[118] Aichen Z., Kun W., Xiaochun S., Lingling T. (2021). LncRNA FGD5-AS1 promotes the malignant phenotypes of ovarian cancer cells via targeting miR-142-5p. Apoptosis.

[119] Zhang Y., Sun L., Chen T., Yue Y., Zhao L., Zhang D. (2023). Lncrna FGFR3-AS1 Is a Prognostic Indicator for Ovarian Cancer and Induces Cell Proliferation and Hinders Apoptosis. Iran. J. Public Health.

[120] Yan H., Li H., Silva M.A., Guan Y., Yang L., Zhu L., Zhang Z., Li G., Ren C. (2019). LncRNA FLVCR1-AS1 mediates miR-513/YAP1 signaling to promote cell progression, migration, invasion and EMT process in ovarian cancer. J. Exp. Clin. Cancer Res..

[121] Gao J., Liu F., Zhao X., Zhang P. (2021). Long non-coding RNA FOXD2-AS1 promotes proliferation, migration and invasion of ovarian cancer cells via regulating the expression of miR-4492. Exp. Ther. Med..

[122] Li Y., Zhu X., Zhang C., Yin Y., Chen L., Liu Y., He A., Xia F. (2023). Long noncoding RNA FTX promotes epithelial-mesenchymal transition of epithelial ovarian cancer through modulating miR-7515/TPD52 and activating Met/Akt/mTOR. Histol. Histopathol..

[123] Li H., Zeng Z., Yang X., Chen Y., He L., Wan T. (2020). LncRNA GClnc1 may contribute to the progression of ovarian cancer by regulating p53 signaling pathway. Eur. J. Histochem..

[124] Wu D., Ke Y., Xiao R., Liu J., Li Q., Wang Y. (2021). Long non-coding RNA GClnc1 knockdown suppresses progression of epithelial ovarian cancer by recruiting FOXC2 to disrupt the NOTCH1/NF-κB/Snail pathway. Exp. Cell Res..

[125] Ma H., Gao L., Yu H., Song X. (2022). Long non-coding RNA H19 correlates with unfavorable prognosis and promotes cell migration and invasion in ovarian cancer. Ginekol. Pol..

[126] Tian X., Zuo X., Hou M., Li C., Teng Y. (2021). LncRNA-H19 regulates chemoresistance to carboplatin in epithelial ovarian cancer through microRNA-29b-3p and STAT3. J. Cancer.

[127] Wang Y., Gao W.J. (2021). Long non-coding RNA-H19 promotes ovarian cancer cell proliferation and migration via the microRNA-140/Wnt1 axis. Kaohsiung J. Med. Sci..

[128] Xu H., Ding Y., Yang X. (2021). Overexpression of Long Noncoding RNA H19 Downregulates miR-140-5p and Activates PI3K/AKT Signaling Pathway to Promote Invasion, Migration and Epithelial-Mesenchymal Transition of Ovarian Cancer Cells. Biomed. Res. Int..

[129] Zhu L., Mei M. (2021). Interference of long non-coding RNA HAGLROS inhibits the proliferation and promotes the apoptosis of ovarian cancer cells by targeting miR-26b-5p. Exp. Ther. Med..

[130] Li X.F., Hu D.M., Zhao Y.X., Zhang L., Jin Y. (2020). Knockdown of lncRNA HCG11 suppresses cell progression in ovarian cancer by modulating miR-144-3p/PBX3. Eur. Rev. Med. Pharmacol. Sci..

[131] Zhang F., Luo B.H., Wu Q.H., Li Q.L., Yang K.D. (2022). LncRNA HCG18 upregulates TRAF4/TRAF5 to facilitate proliferation, migration and EMT of epithelial ovarian cancer by targeting miR-29a/b. Mol. Med..

[132] Wang L., He M., Fu L., Jin Y. (2020). Role of lncRNAHCP5/microRNA-525-5p/PRC1 crosstalk in the malignant behaviors of ovarian cancer cells. Exp. Cell Res..

[133] Shou J., Zhang C., Zheng X., Li Y., Wu P., Chen L., Wei X. (2023). LncRNA HCP5 Facilitates the Progression of Ovarian Cancer by Interacting with the PTBP1 Protein. Biochem. Genet..

[134] Si L., Chen J., Yang S., Liu Z., Chen Y., Peng M., Jia Y. (2020). lncRNA HEIH accelerates cell proliferation and inhibits cell senescence by targeting miR-3619-5p/CTTNBP2 axis in ovarian cancer. Menopause.

[135] Xie W., Wang W., Meng S., Wu X., Liu X., Liu Y., Kang X., Su Y., Lv X., Guo L. (2023). A novel hypoxia-stimulated lncRNA HIF1A-AS3 binds with YBX1 to promote ovarian cancer tumorigenesis by suppressing p21 and AJAP1 transcription. Mol. Carcinog..

[136] Fang W., Xia Y. (2022). LncRNA HLA-F-AS1 attenuates the ovarian cancer development by targeting miR-21-3p/PEG3 axis. Anticancer. Drugs.

[137] Fan L., Lei H., Lin Y., Zhou Z., Li J., Wu A., Shu G., Roger S., Yin G. (2022). Hotair promotes the migration and proliferation in ovarian cancer by miR-222-3p/CDK19 axis. Cell Mol. Life Sci..

[138] Zhou Y., Zhang Y., Shao Y., Yue X., Chu Y., Yang C., Chen D. (2023). LncRNA HOTAIR down-expression inhibits the invasion and tumorigenicity of epithelial ovarian cancer cells by suppressing TGF-β1 and ZEB1. Discov. Oncol..

[139] Jiang J., Wang S., Wang Z., Cai J., Han L., Xie L., Han Q., Wang W., Zhang Y., He X. (2020). HOTAIR promotes paclitaxel resistance by regulating CHEK1 in ovarian cancer. Cancer Chemother. Pharmacol..

[140] Dai Z.Y., Jin S.M., Luo H.Q., Leng H.L., Fang J.D. (2021). LncRNA HOTAIR regulates anoikis-resistance capacity and spheroid formation of ovarian cancer cells by recruiting EZH2 and influencing H3K27 methylation. Neoplasma.

[141] Zhang Y., Ai H., Fan X., Chen S., Wang Y., Liu L. (2020). Knockdown of long non-coding RNA HOTAIR reverses cisplatin resistance of ovarian cancer cells through inhibiting miR-138-5p-regulated EZH2 and SIRT1. Biol. Res..

[142] Zhang Y., Guo J., Cai E., Cai J., Wen Y., Lu S., Li X., Han Q., Jiang J., Li T. (2020). HOTAIR maintains the stemness of ovarian cancer stem cells via the miR-206/TBX3 axis. Exp. Cell Res..

[143] Ye L., Meng X., Xiang R., Li W., Wang J. (2021). Investigating function of long noncoding RNA of HOTAIRM1 in progression of SKOV3 ovarian cancer cells. Drug Dev. Res..

[144] Zhang S., Ma Q., Wu X., Chen P. (2022). LncRNA HOTTIP Promotes Ovarian Cancer Cell Invasion And Metastasis By Stabilizing Hif-1α In The Anoxic Cellular Microenvironment. Acta Endocrinol..

[145] Liu J., Hu H.B., Liu Y.M., Li F.X., Zhang L.P., Liao Z.M. (2020). LncRNA HOTTIP promotes the proliferation and invasion of ovarian cancer cells by activating the MEK/ERK pathway. Mol. Med. Rep..

[146] Tan C., Liu W., Zheng Z.H., Wan X.G. (2021). LncRNA HOTTIP inhibits cell pyroptosis by targeting miR-148a-3p/AKT2 axis in ovarian cancer. Cell Biol. Int..

[147] Dong Y.J., Feng W., Li Y. (2021). HOTTIP-miR-205-ZEB2 Axis Confers Cisplatin Resistance to Ovarian Cancer Cells. Front. Cell Dev. Biol..

[148] Wang Y., Shao W. (2024). LncRNA HOXA-AS2 promotes the progression of epithelial ovarian cancer via the regulation of miR-372. Oncol. Lett..

[149] Eoh K.J., Lee D.W., Nam E.J., Kim J.I., Moon H., Kim S.W., Kim Y.T. (2023). HOXA-AS3 induces tumor progression through the epithelial-mesenchymal transition pathway in epithelial ovarian cancer. Oncol. Rep..

[150] Chen Y., Cui Z., Wu Q., Wang H., Xia H., Sun Y. (2022). Long non-coding RNA HOXA11-AS knockout inhibits proliferation and overcomes drug resistance in ovarian cancer. Bioengineered.

[151] Xu S., Jia G., Zhang H., Wang L., Cong Y., Lv M., Xu J., Ruan H., Jia X., Xu P. (2021). LncRNA HOXB-AS3 promotes growth, invasion and migration of epithelial ovarian cancer by altering glycolysis. Life Sci..

[152] Yang B., Sun L., Liang L. (2021). LncRNA HOXC-AS3 Suppresses the Formation of Mature miR-96 in Ovarian Cancer Cells to Promote Cell Proliferation. Reprod. Sci..

[153] Huang B., Wei M., Hong L. (2021). Long noncoding RNA HULC contributes to paclitaxel resistance in ovarian cancer via miR-137/ITGB8 axis. Open Life Sci..

[154] Zhou J., Xu Y., Wang L., Cong Y., Huang K., Pan X., Liu G., Li W., Dai C., Xu P. (2023). LncRNA IDH1-AS1 sponges miR-518c-5p to suppress proliferation of epithelial ovarian cancer cell by targeting RMB47. J. Biomed. Res..

[155] Liu J., Yan C., Xu S. (2024). LncRNA IL21-AS1 facilitates tumour progression by enhancing CD24-induced phagocytosis inhibition and tumorigenesis in ovarian cancer. Cell Death Dis..

[156] Liu H., Chen R., Kang F., Lai H., Wang Y. (2020). KCNQ1OT1 promotes ovarian cancer progression via modulating MIR-142-5p/CAPN10 axis. Mol. Genet. Genomic Med..

[157] Chen P., Sun L.S., Shen H.M., Qu B. (2022). LncRNA KCNQ1OT1 accelerates ovarian cancer progression via miR-125b-5p/CD147 axis. Pathol. Res. Pract..

[158] He S.L., Chen Y.L., Chen Q.H., Tian Q., Yi S.J. (2022). LncRNA KCNQ1OT1 promotes the metastasis of ovarian cancer by increasing the methylation of EIF2B5 promoter. Mol. Med..

[159] Wu X., Qiu L., Feng H., Zhang H., Yu H., Du Y., Wu H., Zhu S., Ruan Y., Jiang H. (2022). KHDRBS3 promotes paclitaxel resistance and induces glycolysis through modulated MIR17HG/CLDN6 signaling in epithelial ovarian cancer. Life Sci..

[160] Xie X., Wen Q., Yang X., Chen W., Liu Y., Liu W., Zhang T., Xu C., Shi K. (2022). H3K27ac-activated lncRNA KTN1-AS1 aggravates tumor progression by miR-505-3p/ZNF326 axis in ovarian cancer. Ann. Transl. Med..

[161] Gu H., Lin R., Zheng F., Zhang Q. (2021). ELK1 activated-long noncoding RNA LBX2-AS1 aggravates the progression of ovarian cancer through targeting miR-4784/KDM5C axis. J. Mol. Histol..

[162] Zhang Y., Ruan F. (2020). LncRNA LEF1-AS1 Promotes Ovarian Cancer Development Through Interacting with miR-1285-3p. Cancer Manag. Res..

[163] Wang S., Weng W., Chen T., Xu M., Wei P., Li J., Lu L., Wang Y. (2020). LINC00152 Promotes Tumor Progression and Predicts Poor Prognosis by Stabilizing BCL6 From Degradation in the Epithelial Ovarian Cancer. Front. Oncol..

[164] Zou H., Li H. (2019). Knockdown of long non-coding RNA LINC00152 increases cisplatin sensitivity in ovarian cancer cells. Exp. Ther. Med..

[165] Dai L., Niu J., Feng Y. (2020). Knockdown of long non-coding RNA LINC00176 suppresses ovarian cancer progression by BCL3-mediated down-regulation of ceruloplasmin. J. Cell Mol. Med..

[166] Shu C., Liu L., Xue J., Fei J., Chen X., Wang J., Yang X., Peng Q., Zhu W. (2022). The downregulation of LINC00273 inhibits the proliferation, invasion, and migration of ovarian cancer cells in vivo and in vitro. Ann. Transl. Med..

[167] Yang J., Wang W.G., Zhang K.Q. (2020). LINC00452 promotes ovarian carcinogenesis through increasing ROCK1 by sponging miR-501-3p and suppressing ubiquitin-mediated degradation. Aging.

[168] Shu Y., Zhang H., Li J., Shan Y. (2021). LINC00494 Promotes Ovarian Cancer Development and Progression by Modulating NFκB1 and FBXO32. Front. Oncol..

[169] Liu Y., He X., Chen Y., Cao D. (2019). Long non-coding RNA LINC00504 regulates the Warburg effect in ovarian cancer through inhibition of miR-1244. Mol. Cell. Biochem..

[170] Tao L.M., Gong Y.F., Yang H.M., Pei J.H., Zhao X.J., Liu S.S. (2020). LINC00662 promotes glycolysis and cell survival by regulating miR- 375/HIF-1α axis in ovarian cancer. J. Biol. Regul. Homeost. Agents.

[171] Wang S., Liu C., Li Y., Qiao J., Chen X., Bao J., Li R., Xing Y. (2022). LINC00665 affects the malignant biological behavior of ovarian cancer via the miR-148b-3p/KLF5. Syst. Biol. Reprod. Med..

[172] Wang S., Wang Y., Lu J., Wang J. (2022). LncRNA LINC00665 Promotes Ovarian Cancer Cell Proliferation and Inhibits Apoptosis via Targeting miR-181a-5p/FHDC. Appl. Biochem. Biotechnol..

[173] Zhao M.W., Lin C.J., Qiu J.P. (2023). LINC00707 promotes multidrug resistance of ovarian cancer cells by targeting the miR-382-5p/LRRK2 axis. Acta Biochim. Pol..

[174] Qiao Z.W., Jiang Y., Wang L., Wang L., Jiang J., Zhang J.R., Mu P. (2021). LINC00852 promotes the proliferation and invasion of ovarian cancer cells by competitively binding with miR-140-3p to regulate AGTR1 expression. BMC Cancer.

[175] Lin X., Feng D., Li P., Lv Y. (2020). LncRNA LINC00857 regulates the progression and glycolysis in ovarian cancer by modulating the Hippo signaling pathway. Cancer Med..

[176] Xue H., Wu Z., Rao D., Zhuo B., Chen Q. (2020). Long non-coding RNA LINC00858 aggravates the oncogenic phenotypes of ovarian cancer cells through miR-134-5p/RAD18 signaling. Arch. Gynecol. Obstet..

[177] Li P., Huang G. (2022). Long noncoding RNA LINC00858 promotes the progression of ovarian cancer via regulating the miR-134-5p/TRIM44 axis. J. Recept. Signal Transduct. Res..

[178] Yang X., Wu G., Yang F., He L., Xie X., Li L., Yang L., Ma Y., Zhang Q., Chen J. (2021). Elevated LINC00909 Promotes Tumor Progression of Ovarian Cancer via Regulating the miR-23b-3p/MRC2 Axis. Oxid. Med. Cell Longev..

[179] Wang L., Ren C., Xu Y., Yang L., Chen Y., Zhu Y. (2021). The LINC00922 aggravates ovarian cancer progression via sponging miR-361-3p. J. Ovarian Res..

[180] Xie M., Fu Q., Wang P.P., Cui Y.L. (2021). STAT1-Induced Upregulation lncRNA LINC00958 Accelerates the Epithelial Ovarian Cancer Tumorigenesis by Regulating Wnt/β-Catenin Signaling. Dis. Markers.

[181] Xu J., Zhang P., Sun H., Liu Y. (2020). LINC01094/miR-577 axis regulates the progression of ovarian cancer. J. Ovarian Res..

[182] Dong B., Li C., Xu X., Wang Y., Li Y., Li X. (2024). LncRNA LINC01123 promotes malignancy of ovarian cancer by targeting hsa-miR-516b-5p/VEGFA. Genes Genom..

[183] Zhu W., Xiao X., Chen J. (2021). Silencing of the long noncoding RNA LINC01132 alleviates the oncogenicity of epithelial ovarian cancer by regulating the microRNA-431-5p/SOX9 axis. Int. J. Mol. Med..

[184] Liu S., Xi X. (2020). LINC01133 contribute to epithelial ovarian cancer metastasis by regulating miR-495-3p/TPD52 axis. Biochem. Biophys. Res. Commun..

[185] Liu W., Tan S., Bai X., Ma S., Chen X. (2021). Long non-coding RNA LINC01215 promotes epithelial-mesenchymal transition and lymph node metastasis in epithelial ovarian cancer through RUNX3 promoter methylation. Transl. Oncol..

[186] Zhang C., Liu J., Zhang Y., Luo C., Zhu T., Zhang R., Yao R. (2020). LINC01342 promotes the progression of ovarian cancer by absorbing microRNA-30c-2-3p to upregulate HIF3A. J. Cell Physiol..

[187] Li Y., Zhai Y., Chen Y. (2021). GATA1-induced upregulation of LINC01503 promotes carboplatin resistance in ovarian carcinoma by upregulating PD-L1 via sponging miR-766-5p. J. Ovarian Res..

[188] Coan M., Toso M., Cesaratto L., Rigo I., Borgna S., Dalla Pietà A., Zandonà L., Iuri L., Zucchetto A., Piazza C. (2023). LINC01605 Is a Novel Target of Mutant p53 in Breast and Ovarian Cancer Cell Lines. Int. J. Mol. Sci..

[189] Chen J., Li X., Yang L., Zhang J. (2021). Long Non-coding RNA LINC01969 Promotes Ovarian Cancer by Regulating the miR-144-5p/LARP1 Axis as a Competing Endogenous, R.N.A. Front. Cell Dev. Biol..

[190] Li Y., Zhao Z., Sun D., Li Y. (2021). Novel long noncoding RNA LINC02323 promotes cell growth and migration of ovarian cancer via TGF-β receptor 1 by miR-1343-3p. J. Clin. Lab. Anal..

[191] Maleki P., Sheida S.V., Mowla S.J., Soleimani V., Taheri M., Raheb J. (2020). LINK-A long non-coding RNA and VEGF RNA expression in epithelial ovarian cancer patients. Hum. Antibodies.

[192] Jiang X., Cheng Y., He Y., Cong S., Sun L., Wu D., Wu H., Zhang G. (2021). LNC00115 Mediates Cisplatin Resistance by Regulating the miR-7/ERK Signalling Pathway in Ovarian Cancer. Cancer Manag. Res..

[193] Zhou X., Liu M., Deng G., Chen L., Sun L., Zhang Y., Luo C., Tang J. (2021). lncRNA LOC102724169 plus cisplatin exhibit the synergistic anti-tumor effect in ovarian cancer with chronic stress. Mol. Ther. Nucleic Acids.

[194] Yim G.W., Lee D.W., Kim J.I., Kim Y.T. (2022). Long Non-coding RNA LOC285194 Promotes Epithelial Ovarian Cancer Progression via the Apoptosis Signaling Pathway. In Vivo.

[195] Filippov-Levy N., Reich R., Davidson B. (2020). The Biological and Clinical Role of the Long Non-Coding RNA LOC642852 in Ovarian Carcinoma. Int. J. Mol. Sci..

[196] Zhao P., Wang Y., Yu X., Nan Y., Liu S., Li B., Cui Z., Liu Z. (2023). Long noncoding RNA LOC646029 functions as a ceRNA to suppress ovarian cancer progression through the miR-627-3p/SPRED1 axis. Front. Med..

[197] Xue F., Xu Y.H., Shen C.C., Qin Z.L., Zhou H.B. (2020). Non-coding RNA LOXL1-AS1 exhibits oncogenic activity in ovarian cancer via regulation of miR-18b-5p/VMA21 axis. Biomed. Pharmacother..

[198] Liu H.Z., Liu G.Y., Pang W.W., Zhang H., Zeng Z.J., Wang H.J. (2020). LncRNA LUCAT1 promotes proliferation of ovarian cancer cells by regulating miR-199a-5p expression. Eur. Rev. Med. Pharmacol. Sci..

[199] Bai Y., Ren C., Wang B., Xue J., Li F., Liu J., Yang L. (2021). LncRNA MAFG-AS1 promotes the malignant phenotype of ovarian cancer by upregulating NFKB1-dependent IGF1. Cancer Gene Ther..

[200] Mao T.L., Fan M.H., Dlamini N., Liu C.L. (2021). LncRNA MALAT1 Facilitates Ovarian Cancer Progression through Promoting Chemoresistance and Invasiveness in the Tumor Microenvironment. Int. J. Mol. Sci..

[201] Pei C., Gong X., Zhang Y. (2020). LncRNA MALAT-1 promotes growth and metastasis of epithelial ovarian cancer via sponging microrna-22. Am. J. Transl. Res..

[202] Zhu Y., Yang L., Wang J., Li Y., Chen Y. (2022). SP1-induced lncRNA MCF2L-AS1 promotes cisplatin resistance in ovarian cancer by regulating IGF2BP1/IGF2/MEK/ERK axis. J. Gynecol. Oncol..

[203] Zhou S., Xu A., Song T., Gao F., Sun H., Kong X. (2020). lncRNA MIAT Regulates Cell Growth, Migration, and Invasion Through Sponging miR-150-5p in Ovarian Cancer. Cancer Biother. Radiopharm..

[204] Fan Y., Wang L., Han X.C., Ma H.Y., Zhang N., Zhe L. (2020). LncRNA MIF-AS1 aggravates the progression of ovarian cancer by sponging miRNA-31-5p. Eur. Rev. Med. Pharmacol. Sci..

[205] Liu P., Huang H., Qi X., Bian C., Cheng M., Liu L., Xue L., Zhao X., Yi T., Quan Y. (2021). Hypoxia-Induced LncRNA-MIR210HG Promotes Cancer Progression By Inhibiting HIF-1α Degradation in Ovarian Cancer. Front. Oncol..

[206] Zhu L., Wang A., Gao M., Duan X., Li Z. (2020). LncRNA MIR4435-2HG triggers ovarian cancer progression by regulating miR-128-3p/CKD14 axis. Cancer Cell Int..

[207] Wu A., Liu J., Zhang X., Niu C., Shu G., Yin G. (2022). Comprehensive network analysis of dysregulated genes revealed MNX1-AS1/hsa-miR-4697-3p/HOXB13 axis in ovarian cancer chemotherapy response. Cancer Sci..

[208] Shen Y., Lv M., Fang Y., Lu J., Wu Y. (2021). LncRNA MNX1-AS1 promotes ovarian cancer process via targeting the miR-744-5p/SOX12 axis. J. Ovarian Res..

[209] Zhang G., Zheng D., Chen X., Li L., Yu J. (2021). miR-152-mediated MKK7 downregulation is attenuated by MYCNOS in ovarian adenocarcinoma. Oncol. Lett..

[210] Wang S., Zheng Q., Wang J., Chen S., Chen L. (2023). Long non-coding RNA MYU promotes ovarian cancer cell proliferation by sponging miR-6827-5p and upregulating HMGA1. Pathol. Oncol. Res..

[211] Chang H., Li B., Zhang X., Meng X. (2020). NCK1-AS1 promotes NCK1 expression to facilitate tumorigenesis and chemo-resistance in ovarian cancer. Biochem. Biophys. Res. Commun..

[212] Luo X., Wei Q., Jiang X., Chen N., Zuo X., Zhao H., Liu Y., Liu X., Xie L., Yang Y. (2024). CSTF3 contributes to platinum resistance in ovarian cancer through alternative polyadenylation of lncRNA NEAT1 and generating the short isoform NEAT1_1. Cell Death Dis..

[213] Liu Y., Li Y., Wu Y., Zhao Y., Hu X., Sun C. (2023). The long non-coding RNA NEAT1 promotes the progression of human ovarian cancer by targeting miR-214-3p and regulating angiogenesis. J. Ovarian Res..

[214] Zhu M., Yang L., Wang X. (2020). NEAT1 Knockdown Suppresses the Cisplatin Resistance in Ovarian Cancer by Regulating miR-770-5p/PARP1 Axis. Cancer Manag. Res..

[215] Yuan J., Yi K., Yang L. (2021). LncRNA NEAT1 promotes proliferation of ovarian cancer cells and angiogenesis of co-incubated human umbilical vein endothelial cells by regulating FGF9 through sponging miR-365: An experimental study. Medicine.

[216] Xu H., Sun X., Huang Y., Si Q., Li M. (2020). Long non-coding RNA NEAT1 modifies cell proliferation, colony formation, apoptosis, migration and invasion via the miR-4500/BZW1 axis in ovarian cancer. Mol. Med. Rep..

[217] Yin L., Wang Y. (2021). Long non-coding RNA NEAT1 facilitates the growth, migration, and invasion of ovarian cancer cells via the let-7 g/MEST/ATGL axis. Cancer Cell Int..

[218] Luo M., Zhang L., Yang H., Luo K., Qing C. (2020). Long non-coding RNA NEAT1 promotes ovarian cancer cell invasion and migration by interacting with miR-1321 and regulating tight junction protein 3 expression. Mol. Med. Rep..

[219] Xu C., Zhu L.X., Sun D.M., Yao H., Han D.X. (2020). Regulatory mechanism of lncRNA NORAD on proliferation and invasion of ovarian cancer cells through miR-199a-3p. Eur. Rev. Med. Pharmacol. Sci..

[220] Chen Q., Xie J., Yang Y. (2022). Long non-coding RNA NRSN2-AS1 facilitates tumorigenesis and progression of ovarian cancer via miR-744-5p/PRKX axis. Biol. Reprod..

[221] Wu Y.B., Li S.Y., Liu J.Y., Xue J.J., Xu J.F., Chen T., Cao T.Y., Zhou H., Wu T.T., Dong C.L. (2023). Long non-coding RNA NRSN2-AS1 promotes ovarian cancer progression through targeting PTK2/β-catenin pathway. Cell Death Dis..

[222] Wang Y., Li L., Zhang X., Zhao X. (2022). Long non-coding RNA OIP5-AS1 suppresses microRNA-92a to augment proliferation and metastasis of ovarian cancer cells through upregulating ITGA6. J. Ovarian Res..

[223] Liu Q.Y., Jiang X.X., Tian H.N., Guo H.L., Guo H., Guo Y. (2020). Long non-coding RNA OIP5-AS1 plays an oncogenic role in ovarian cancer through targeting miR-324-3p/NFIB axis. Eur. Rev. Med. Pharmacol. Sci..

[224] Guo L., Chen J., Liu D., Liu L. (2020). OIP5-AS1/miR-137/ZNF217 Axis Promotes Malignant Behaviors in Epithelial Ovarian Cancer. Cancer Manag. Res..

[225] Jiang X., Ye Z., Jiang Y., Yu W., Fang Q. (2020). LncRNA OIP5-AS1 upregulates snail expression by sponging miR-34a to promote ovarian carcinoma cell invasion and migration. Biol. Res..

[226] Liu Y., Fu X., Wang X., Liu Y., Song X. (2021). Long non-coding RNA OIP5-AS1 facilitates the progression of ovarian cancer via the miR-128-3p/CCNG1 axis. Mol. Med. Rep..

[227] Guo F., Du J., Liu L., Gou Y., Zhang M., Sun W., Yu H., Fu X. (2021). lncRNA OR3A4 Promotes the Proliferation and Metastasis of Ovarian Cancer Through KLF6 Pathway. Front. Pharmacol..

[228] Wang J., Han Y., Zhang T., Li J., Xu B. (2023). LncRNA PART1Regulates Ovarian Carcinoma Development via the miR-150-5p/MYB Axis. Front. Biosci..

[229] Li H., Lei Y., Li S., Li F., Lei J. (2022). LncRNA PART1 Stimulates the Development of Ovarian Cancer by Up-regulating RACGAP1 and RRM2. Reprod. Sci..

[230] Li B., Lou G., Zhang J., Cao N., Yu X. (2022). Repression of lncRNA PART1 attenuates ovarian cancer cell viability, migration and invasion through the miR-503-5p/FOXK1 axis. BMC Cancer.

[231] Liu Y., Zong Z.H., Guan X., Wang L.L., Zhao Y. (2017). The role of long non-coding RNA PCA3 in epithelial ovarian carcinoma tumorigenesis and progression. Gene.

[232] He Y., Wei L., Zhang S., Liu H., Fang F., Li Y. (2020). LncRNA PLAC2 Positively Regulates CDK2 to Promote Ovarian Carcinoma Cell Proliferation. Cancer Manag. Res..

[233] Zhao Y., Hong L. (2021). lncRNA-PRLB Confers Paclitaxel Resistance of Ovarian Cancer Cells by Regulating RSF1/NF-κB Signaling Pathway. Cancer Biother. Radiopharm..

[234] Qi X., Chen D., Yu W., Wang L., Liu L., Tao X. (2022). Long non-coding RNA PRNCR1 promotes ovarian cancer cell proliferation, migration and invasion by targeting the miR-653-5p/ELF2 axis. Mol. Cell Biochem..

[235] Wang H., Zhou Y., Zhang S., Qi Y.A., Wang M. (2022). PRPF6 promotes metastasis and paclitaxel resistance of ovarian cancer via SNHG16/CEBPB/GATA3 axis. Oncol. Res..

[236] Xu Z., Jin H., Duan X., Liu H., Zhao X., Fan S., Wang Y., Yao T. (2021). LncRNA PSMA3-AS1 promotes cell proliferation, migration, and invasion in ovarian cancer by activating the PI3K/Akt pathway via the miR-378a-3p/GALNT3 axis. Environ. Toxicol..

[237] Liang H., Yu M., Yang R., Zhang L., Zhang L., Zhu D., Luo H., Hong Y., Yu T., Sun J. (2019). A PTAL-miR-101-FN1 Axis Promotes EMT and Invasion-Metastasis in Serous Ovarian Cancer. Mol. Ther. Oncolytics.

[238] Zhang R., He T., Shi H., Yuan C., Wei F., Liu Z., Wang W. (2021). Disregulations of PURPL and MiR-338-3p Could Serve As Prognosis Biomarkers for Epithelial Ovarian Cancer. J. Cancer.

[239] Dong L., Wang H., Gao Y., Wang S., Wang W. (2022). Long non-coding RNA PVT1 promotes the proliferation, migration and EMT process of ovarian cancer cells by regulating CTGF. Oncol. Lett..

[240] Wu Y., Gu W., Han X., Jin Z. (2021). LncRNA PVT1 promotes the progression of ovarian cancer by activating TGF-β pathway via miR-148a-3p/AGO1 axis. J. Cell Mol. Med..

[241] Li M., Chi C., Zhou L., Chen Y., Tang X. (2021). Circular PVT1 regulates cell proliferation and invasion via miR-149-5p/FOXM1 axis in ovarian cancer. J. Cancer.

[242] Yi K., Hou M., Yuan J., Yang L., Zeng X., Xi M., Chen J. (2020). LncRNA PVT1 epigenetically stabilizes and post-transcriptionally regulates FOXM1 by acting as a microRNA sponge and thus promotes malignant behaviors of ovarian cancer cells. Am. J. Transl. Res..

[243] Qu C., Dai C., Guo Y., Qin R., Liu J. (2020). Long non-coding RNA PVT1-mediated miR-543/SERPINI1 axis plays a key role in the regulatory mechanism of ovarian cancer. Biosci. Rep..

[244] Tabury K., Monavarian M., Listik E., Shelton A.K., Choi A.S., Quintens R., Arend R.C., Hempel N., Miller C.R., Györrfy B. (2022). PVT1 is a stress-responsive lncRNA that drives ovarian cancer metastasis and chemoresistance. Life Sci. Alliance.

[245] Zhao L., Huang J., Liu W., Su X., Zhao B., Wang X., He X. (2024). Long non-coding RNA RAD51-AS1 promotes the tumorigenesis of ovarian cancer by elevating EIF5A2 expression. J. Cancer Res. Clin. Oncol..

[246] Luo J., Zhang Y., Zheng T., Jing Y., Cao R., Wu M., Fan D., Tao Y., Zhao M. (2023). Application of long non-coding RNA RBAT1 in improving diagnosis and prognosis of ovarian carcinoma. Anticancer. Drugs.

[247] Cui S. (2022). METTL3-mediated m6A modification of lnc RNA RHPN1-AS1 enhances cisplatin resistance in ovarian cancer by activating PI3K/AKT pathway. J. Clin. Lab. Anal..

[248] Zhao L., Liu T., Zhang X., Zuo D., Liu C. (2020). lncRNA RHPN1-AS1 Promotes Ovarian Cancer Growth and Invasiveness Through Inhibiting miR-1299. Onco Targets Ther..

[249] Zhao J., Yang T., Ji J., Zhao F., Li C., Han X. (2021). RHPN1-AS1 promotes cell proliferation and migration via miR-665/Akt3 in ovarian cancer. Cancer Gene Ther..

[250] Wang J., Ding W., Xu Y., Tao E., Mo M., Xu W., Cai X., Chen X., Yuan J., Wu X. (2020). Long non-coding RNA RHPN1-AS1 promotes tumorigenesis and metastasis of ovarian cancer by acting as a ceRNA against miR-596 and upregulating LETM1. Aging.

[251] Cui S., Li F. (2021). RHPN1-AS1 promotes ovarian carcinogenesis by sponging miR-6884-5p thus releasing TOP2A mRNA. Oncol. Rep..

[252] Cui S., Li C. (2021). RHPN1-AS1 promotes ovarian carcinogenesis by sponging miR-485-5p and releasing TPX2 mRNA. Oncol. Rep..

[253] Li L., Zeng S., Guo L., Huang P., Xi J., Feng J., Li Q., Li Y., Xiao X., Yan R. (2022). Long Noncoding RNA RMRP Contributes to Paclitaxel Sensitivity of Ovarian Cancer by Regulating miR-580-3p/MICU1 Signaling. J. Oncol..

[254] Sun P., Bao A., Hua X., Cao J., Ding Y. (2022). RP5-1148A21.3 (lncRP5) exerts oncogenic function in human ovarian carcinoma. Acta Biochim. Biophys. Sin..

[255] Luan A.A., Hou L.L., Zhang F.Y. (2022). Silencing of SBF2-AS1 inhibits cell growth and invasion by sponging microRNA-338-3p in serous ovarian carcinoma. Kaohsiung J. Med. Sci..

[256] Song R., Liu Z., Lu L., Liu F., Zhang B. (2020). Long Noncoding RNA SCAMP1 Targets miR-137/CXCL12 Axis to Boost Cell Invasion and Angiogenesis in Ovarian Cancer. DNA Cell Biol..

[257] Zhao H., Wang A., Zhang Z. (2020). LncRNA SDHAP1 confers paclitaxel resistance of ovarian cancer by regulating EIF4G2 expression via miR-4465. J. Biochem..

[258] Wu Y., Zhu B., Yan Y., Bai S., Kang H., Zhang J., Ma W., Gao Y., Hui B., Li R. (2021). Long non-coding RNA SNHG1 stimulates ovarian cancer progression by modulating expression of miR-454 and ZEB1. Mol. Oncol..

[259] Abildgaard C., do Canto L.M., Rainho C.A., Marchi F.A., Calanca N., Waldstrøm M., Steffensen K.D., Rogatto S.R. (2022). The Long Non-Coding RNA SNHG12 as a Mediator of Carboplatin Resistance in Ovarian Cancer via Epigenetic Mechanisms. Cancers.

[260] Zhang J., Wang L., Jiang J., Qiao Z. (2020). The lncRNA SNHG15/miR-18a-5p axis promotes cell proliferation in ovarian cancer through activating Akt/mTOR signaling pathway. J. Cell Biochem..

[261] Wang Y., Ding M., Yuan X., Jiao R., Zhu D., Huang W., Deng W., Liu Y. (2021). lncRNA SNHG15 Promotes Ovarian Cancer Progression through Regulated CDK6 via Sponging miR-370-3p. Biomed. Res. Int..

[262] Liang H., Geng S., Wang Y., Fang Q., Xin Y., Li Y. (2024). Tumour-derived exosome SNHG17 induced by oestrogen contributes to ovarian cancer progression via the CCL13-CCR2-M2 macrophage axis. J. Cell Mol. Med..

[263] Pan X., Guo Z., Chen Y., Zheng S., Peng M., Yang Y., Wang Z. (2020). STAT3-Induced lncRNA SNHG17 Exerts Oncogenic Effects on Ovarian Cancer through Regulating CDK6. Mol. Ther. Nucleic. Acids..

[264] Wang W., Yu S., Li W., Hu H., Zou G. (2022). Silencing of lncRNA SNHG17 inhibits the tumorigenesis of epithelial ovarian cancer through regulation of miR-485-5p/AKT1 axis. Biochem. Biophys. Res. Commun..

[265] Yang Q., Dong Y.J. (2021). LncRNA SNHG20 promotes migration and invasion of ovarian cancer via modulating the microRNA-148a/ROCK1 axis. J. Ovarian Res..

[266] Xing X., An M., Chen T. (2021). LncRNA SNHG20 promotes cell proliferation and invasion by suppressing miR-217 in ovarian cancer. Genes. Genom..

[267] Wang D., Li Z., Li H., Lu J., Qin Q. (2021). Long non-coding RNA SNHG20 promotes ovarian cancer development by targeting microRNA-338-3p to regulate MCL1 expression. Oncol. Lett..

[268] Guan N., Zheng H., Wu X., Xie L., Tong X. (2021). SP1-Regulated Non-Coding RNA SNHG22 Promotes Ovarian Cancer Growth and Glycolysis. Cancer Manag. Res..

[269] Liu Y., Xu B., Liu M., Qiao H., Zhang S., Qiu J., Ying X. (2021). Long non-coding RNA SNHG25 promotes epithelial ovarian cancer progression by up-regulating COMP. J. Cancer.

[270] Zhang L., Li G., Wang X., Zhang Y., Huang X., Wu H. (2021). lncRNA SNHG3 acts as oncogene in ovarian cancer through miR-139-5p and Notch1. Oncol. Lett..

[271] Liu E.L., Zhou Y.X., Li J., Zhang D.H., Liang F. (2020). Long-Chain Non-Coding RNA SNHG3 Promotes the Growth of Ovarian Cancer Cells by Targeting miR-339-5p/TRPC3 Axis. Onco Targets Ther..

[272] Liu C., Zhao S., Lv Z.X., Zhao X.J. (2023). Promoting action of long non-coding RNA small nucleolar RNA host gene 4 in ovarian cancer. Acta Biochim. Pol..

[273] Su M., Huang P., Li Q. (2023). Long noncoding RNA SNHG6 promotes the malignant phenotypes of ovarian cancer cells via miR-543/YAP1 pathway. Heliyon.

[274] Zhang J., Zhang R., Ye Y. (2021). Long non-coding RNA (LncRNA) SNHG7/ Eukaryotic translation initiation factor 4 gamma 2 (EIF4G2) involves in the malignant events of ovarian cancer cells with paclitaxel resistant. Bioengineered.

[275] Bai Z., Wu Y., Bai S., Yan Y., Kang H., Ma W., Zhang J., Gao Y., Hui B., Ma H. (2020). Long non-coding RNA SNGH7 Is activated by SP1 and exerts oncogenic properties by interacting with EZH2 in ovarian cancer. J. Cell Mol. Med..

[276] Miao W., Lu T., Liu X., Yin W., Zhang H. (2020). LncRNA SNHG8 induces ovarian carcinoma cells cellular process and stemness through Wnt/β-catenin pathway. Cancer Biomark..

[277] Wang C., Wang J., Shen X., Li M., Yue Y., Cheng X., Lu W., Wang X., Xie X. (2021). LncRNA SPOCD1-AS from ovarian cancer extracellular vesicles remodels mesothelial cells to promote peritoneal metastasis via interacting with G3BP1. J. Exp. Clin. Cancer Res..

[278] Kim L.K., Park S.A., Yang Y., Kim Y.T., Heo T.H., Kim H.J. (2021). LncRNA SRA mediates cell migration, invasion, and progression of ovarian cancer via NOTCH signaling and epithelial-mesenchymal transition. Biosci. Rep..

[279] Qiu J.J., Lin X.J., Tang X.Y., Zheng T.T., Zhang X.Y., Hua K.Q. (2020). Long noncoding RNA TC0101441 induces epithelial-mesenchymal transition in epithelial ovarian cancer metastasis by downregulating KiSS1. Int. J. Cancer.

[280] Ge J., Han T., Shan L., Na J., Li Y., Wang J. (2020). Long non-coding RNA THOR promotes ovarian Cancer cells progression via IL-6/STAT3 pathway. J. Ovarian Res..

[281] Mu Q., Wang X., Huang K., Xia B., Bi S., Kong Y. (2024). THUMPD3-AS1 inhibits ovarian cancer cell apoptosis through the miR-320d/ARF1 axis. FASEB J..

[282] Xu Q., Lin Y.B., Li L., Liu J. (2020). LncRNA TLR8-AS1 promotes metastasis and chemoresistance of ovarian cancer through enhancing TLR8 mRNA stability. Biochem. Biophys. Res. Commun..

[283] Zhao H., Ding F., Zheng G. (2020). LncRNA TMPO-AS1 promotes LCN2 transcriptional activity and exerts oncogenic functions in ovarian cancer. FASEB J..

[284] Li H., Zhou Y., Cheng H., Tian J., Yang S. (2020). Roles of a TMPO-AS1/microRNA-200c/TMEFF2 ceRNA network in the malignant behaviors and 5-FU resistance of ovarian cancer cells. Exp. Mol. Pathol..

[285] Liu Y., Li L., Wang X., Wang P., Wang Z. (2020). LncRNA TONSL-AS1 regulates miR-490-3p/CDK1 to affect ovarian epithelial carcinoma cell proliferation. J. Ovarian Res..

[286] Ding Y., Tan X., Abasi A., Dai Y., Wu R., Zhang T., Li K., Yan M., Huang X. (2021). LncRNA TRPM2-AS promotes ovarian cancer progression and cisplatin resistance by sponging miR-138-5p to release SDC3 mRNA. Aging.

[287] Dai T., Liang J., Liu W., Zou Y., Niu F., Li M., Zhang H., Li C., Fan M., Cui G. (2021). The miRNA mir-582-3p suppresses ovarian cancer progression by targeting AKT/MTOR signaling via lncRNA TUG1. Bioengineered.

[288] Gu L., Li Q., Liu H., Lu X., Zhu M. (2020). Long Noncoding RNA TUG1 Promotes Autophagy-Associated Paclitaxel Resistance by Sponging miR-29b-3p in Ovarian Cancer Cells. Onco Targets Ther..

[289] Pei Y., Li K., Lou X., Wu Y., Dong X., Wang W., Li N., Zhang D., Cui W. (2020). miR-1299/NOTCH3/TUG1 feedback loop contributes to the malignant proliferation of ovarian cancer. Oncol. Rep..

[290] Zhan F.L., Chen C.F., Yao M.Z. (2020). LncRNA TUG1 facilitates proliferation, invasion and stemness of ovarian cancer cell via miR-186-5p/ZEB1 axis. Cell Biochem. Funct..

[291] Sonobe R., Yang P., Suzuki M.M., Shinjo K., Iijima K., Nishiyama N., Miyata K., Kataoka K., Kajiyama H., Kondo Y. (2024). Long noncoding RNA TUG1 promotes cisplatin resistance in ovarian cancer via upregulation of DNA polymerase eta. Cancer Sci..

[292] Wambecke A., Ahmad M., Morice P.M., Lambert B., Weiswald L.B., Vernon M., Vigneron N., Abeilard E., Brotin E., Figeac M. (2021). The lncRNA ‘UCA1’ modulates the response to chemotherapy of ovarian cancer through direct binding to miR-27a-5p and control of UBE2N levels. Mol. Oncol..

[293] Li Z.Y., Wang X.L., Dang Y., Zhu X.Z., Zhang Y.H., Cai B.X., Zheng L. (2020). Long non-coding RNA UCA1 promotes the progression of paclitaxel resistance in ovarian cancer by regulating the miR-654-5p/SIK2 axis. Eur. Rev. Med. Pharmacol. Sci..

[294] Wang H., Su H., Tan Y. (2020). UNC5B-AS1 promoted ovarian cancer progression by regulating the H3K27me on NDRG2 via EZH2. Cell Biol. Int..

[295] Guo B., Yu L., Sun Y., Yao N., Ma L. (2020). Long Non-Coding RNA USP2-AS1 Accelerates Cell Proliferation and Migration in Ovarian Cancer by Sponging miR-520d-3p and Up-Regulating KIAA1522. Cancer Manag. Res..

[296] Xiong J., Chen J., Sun X., Zhao R., Gao K. (2023). Prognostic role of long non-coding RNA USP30-AS1 in ovarian cancer: Insights into immune cell infiltration in the tumor microenvironment. Aging.

[297] Wu Y., Wang T., Xia L., Zhang M. (2021). LncRNA WDFY3-AS2 promotes cisplatin resistance and the cancer stem cell in ovarian cancer by regulating hsa-miR-139-5p/SDC4 axis. Cancer Cell Int..

[298] Xia X., Li Z., Li Y., Ye F., Zhou X. (2022). LncRNA XIST promotes carboplatin resistance of ovarian cancer through activating autophagy via targeting miR-506-3p/FOXP1 axis. J. Gynecol. Oncol..

[299] Meng Q., Wang N., Duan G. (2021). Long. non-coding RNA XIST regulates ovarian cancer progression via modulating miR-335/BCL2L2 axis. World J. Surg. Oncol..

[300] Huang K.C., Rao P.H., Lau C.C., Heard E., Ng S.K., Brown C., Mok S.C., Berkowitz R.S., Ng S.W. (2002). Relationship of XIST expression and responses of ovarian cancer to chemotherapy. Mol. Cancer Ther..

[301] Jiang R., Zhang H., Zhou J., Wang J., Xu Y., Zhang H., Gu Y., Fu F., Shen Y., Zhang G. (2021). Inhibition of long non-coding RNA XIST upregulates microRNA-149-3p to repress ovarian cancer cell progression. Cell Death Dis..

[302] Dai C., Xu P., Liu S., Xu S., Xu J., Fu Z., Cao J., Lv M., Zhou J., Liu G. (2021). Long noncoding RNA ZEB1-AS1 affects paclitaxel and cisplatin resistance by regulating MMP19 in epithelial ovarian cancer cells. Arch. Gynecol. Obstet..

[303] Chen Z.J., Zhang Z., Xie B.B., Zhang H.Y. (2016). Clinical significance of up-regulated lncRNA NEAT1 in the prognosis of ovarian cancer. Eur. Rev. Med. Pharmacol. Sci..

[304] Zuo K., Zhao Y., Zheng Y., Chen D., Liu X., Du S., Liu Q. (2019). Long non-coding RNA XIST promotes malignant behavior of epithelial ovarian cancer. Onco Targets Ther..

[305] Wang C., Qi S., Xie C., Li C., Wang P., Liu D. (2018). Upregulation of long non-coding RNA XIST has anticancer effects on epithelial ovarian cancer cells through inverse downregulation of hsa-miR-214-3p. J. Gynecol. Oncol..

[306] Chen C., Liu W.R., Zhang B., Zhang L.M., Li C.G., Liu C., Zhang H., Huo Y.S., Ma Y.C., Tian P.F. (2020). LncRNA H19 downregulation confers erlotinib resistance through upregulation of PKM2 and phosphorylation of AKT in EGFR-mutant lung cancers. Cancer Lett..

[307] Li J., Huang Y., Deng X., Luo M., Wang X., Hu H., Liu C., Zhong M. (2018). Long noncoding RNA H19 promotes transforming growth factor-beta-induced epithelial-mesenchymal transition by acting as a competing endogenous RNA of miR-370-3p in ovarian cancer cells. OncoTargets Ther..

[308] Wu Y., Zhou Y., He J., Sun H., Jin Z. (2019). Long non-coding RNA H19 mediates ovarian cancer cell cisplatin-resistance and migration during EMT. Int. J. Clin. Exp. Pathol..

[309] An J., Lv W., Zhang Y. (2017). LncRNA NEAT1 contributes to paclitaxel resistance of ovarian cancer cells by regulating ZEB1 expression via miR-194. OncoTargets Ther..

[310] Wu L., Wang X., Guo Y. (2017). Long non-coding RNA MALAT1 is upregulated and involved in cell proliferation, migration and apoptosis in ovarian cancer. Exp. Ther. Med..

[311] Qiu J.J., Lin X.J., Tang X.Y., Zheng T.T., Lin Y.Y., Hua K.Q. (2018). Exosomal Metastasis-Associated Lung Adenocarcinoma Transcript 1 Promotes Angiogenesis and Predicts Poor Prognosis in Epithelial Ovarian Cancer. Int. J. Biol. Sci..

[312] Qiu Y.R., Zhao M.Y., Sun L., Yang B.C., Hei K.W., Du X., Li Y.M. (2017). Expression of IncRNA UCA1 in ovarian cancer and its clinical significance. Eur. J. Gynaecol. Oncol..

[313] Wang F., Zhou J., Xie X., Hu J., Chen L., Hu Q., Guo H., Yu C. (2015). Involvement of SRPK1 in cisplatin resistance related to long non-coding RNA UCA1 in human ovarian cancer cells. Neoplasma..

[314] Li Z., Niu H., Qin Q., Yang S., Wang Q., Yu C., Wei Z., Jin Z., Wang X., Yang A. (2019). lncRNA UCA1 Mediates Resistance to Cisplatin by Regulating the miR-143/FOSL2-Signaling Pathway in Ovarian Cancer. Mol. Ther. Nucleic Acids.

[315] Qiu J.J., Lin Y.Y., Ye L.C., Ding J.X., Feng W.W., Jin H.Y., Zhang Y., Li Q., Hua K.Q. (2014). Overexpression of long non-coding RNA HOTAIR predicts poor patient prognosis and promotes tumor metastasis in epithelial ovarian cancer. Gynecol. Oncol..

[316] Wang Y., Wang H., Song T., Zou Y., Jiang J., Fang L., Li P. (2015). HOTAIR is a potential target for the treatment of cisplatin-resistant ovarian cancer. Mol. Med. Rep..

[317] Teschendorff A.E., Lee S.H., Jones A., Fiegl H., Kalwa M., Wagner W., Chindera K., Evans I., Dubeau L., Orjalo A. (2015). HOTAIR and its surrogate DNA methylation signature indicate carboplatin resistance in ovarian cancer. Genome Med..

[318] Wu H., Shang X., Shi Y., Yang Z., Zhao J., Yang M., Li Y., Xu S. (2016). Genetic variants of lncRNA HOTAIR and risk of epithelial ovarian cancer among Chinese women. Oncotarget.

[319] Domcke S., Sinha R., Levine D.A., Sander C., Schultz N. (2013). Evaluating cell lines as tumor models by comparison of genomic profiles. Nat. Commun..

[320] Croft P.K., Sharma S., Godbole N., Rice G.E., Salomon C. (2021). Ovarian-Cancer-Associated Extracellular Vesicles: Microenvironmental Regulation and Potential Clinical Applications. Cells.

[321] Arriaga-Canon C., Contreras-Espinosa L., Aguilar-Villanueva S., Bargalló-Rocha E., García-Gordillo J.A., Cabrera-Galeana P., Castro-Hernández C., Jiménez-Trejo F., Herrera L.A. (2023). The Clinical Utility of lncRNAs and Their Application as Molecular Biomarkers in Breast Cancer. Int. J. Mol. Sci..

